# Mindfulness-based cognitive therapy versus treatment as usual after non-remission with NHS Talking Therapies high-intensity psychological therapy for depression: a UK-based clinical effectiveness and cost-effectiveness randomised, controlled, superiority trial

**DOI:** 10.1016/S2215-0366(25)00105-1

**Published:** 2025-06

**Authors:** Thorsten Barnhofer, Barnaby D Dunn, Clara Strauss, Florian A Ruths, Barbara Barrett, Mary Ryan, Asha Ladwa, Frances Stafford, Roberta Fichera, Hannah Baber, Ailis McGuinness, Isabella Metcalfe, Daniel K Y Kan, Joanna Pooley, Delilah Harding, Emma Tassie, James Carson, Shelley Rhodes, Allan H Young, James Connors, Fiona C Warren

**Affiliations:** a Department of Psychological Interventions, School of Psychology, University of Surrey, Guildford, UK; b Mood Disorders Centre, University of Exeter, Exeter, UK; c Exeter Clinical Trials Unit, University of Exeter, Exeter, UK; d Sussex Partnership NHS Foundation Trust, Hove, UK; e South London and Maudsley NHS Foundation Trust, London, UK; f King's Health Economics, King's College London, London, UK; g National Collaborating Centre for Mental Health, Royal College of Psychiatrists, London, UK; h Devon Partnership NHS Trust, Exeter, UK; i Division of Psychiatry, Imperial College London, London, UK; j School of Psychology, University of Sussex, Brighton, UK

## Abstract

**Background:**

Non-remission after psychological therapy for major depressive disorder is common, yet there are no established further-line treatments. In the UK National Health Service (NHS) Talking Therapies programme, about 50% of patients with depression who come to the end of the stepped care pathway do not show remission of symptoms. We aimed to investigate whether mindfulness-based cognitive therapy (MBCT) can improve clinical outcomes and whether the additional financial cost is worthwhile.

**Methods:**

We conducted a parallel, randomised, controlled, superiority trial in three sites in the UK (Devon, London, and Sussex). Patients with current major depressive disorder whose symptoms had not reached remission (assessed as Patient Health Questionnaire-9 [PHQ-9] score ≥10) after an adequate dose of NHS Talking Therapies high-intensity therapy (≥12 sessions) were recruited from 20 NHS Talking Therapies services. Participants were allocated through remote random assignment (1:1) to MBCT plus treatment as usual or treatment as usual alone at the UK Clinical Research Collaboration-registered Exeter Clinical Trials Unit with minimisation on depression severity (PHQ-9 score <19 *vs* ≥19), antidepressant use at baseline (yes *vs* no), and recruitment site (Devon *vs* London *vs* Sussex). MBCT was delivered via videoconference and comprised an individual orientation session and eight weekly group sessions. The primary clinical outcome was reduction in depression symptomatology at 34 weeks after randomisation, using the PHQ-9. Cost-effectiveness was evaluated in terms of costs to primary, secondary, and tertiary health and social care services collected using the Adult Service Use Schedule and quality-adjusted life-years (QALYs) via health utilities derived from the EQ-5D. Primary outcome analyses were masked in the intention-to-treat population using observed data only. Lived experience experts were integral to all stages of this research. The trial was prospectively registered with ISRCTN, ISRCTN17755571.

**Findings:**

Between April 20, 2021, and Jan 24, 2023, we enrolled 234 eligible participants, 166 (71%) of whom identified as women, 65 (28%) as men, one (<1%) as other, and two (1%) preferred not to say. The mean age was 42·5 years (SD 13·9). 201 (86%) of 234 participants were White. 118 participants were assigned to MBCT plus treatment as usual and 116 to treatment as usual alone, 101 and 102 of whom completed the final follow-up, respectively. At 34 weeks after randomisation, the MBCT plus treatment as usual group had significantly lower levels of depression symptomatology than the treatment as usual alone group (adjusted between-group difference –2·49, 95% CI –3·89 to –1·09; p=0·0006; Cohen's d –0·41, 95% CI –0·67 to –0·15). Utility scores were higher and costs were lower in the MBCT group (adjusted mean cost difference –£245·23, 95% CI –581·92 to 91·46; p=0·15) over the course of the study. The MBCT plus treatment as usual group had an estimated 99% chance of being cost-effective at the £20 000 per QALY threshold. Bootstrapped mean differences in costs and QALYs indicated a 91% probability of MBCT plus treatment as usual being less costly and more effective than treatment as usual alone for all values a decision maker might be willing to pay for an improvement in QALYs. We observed no trial or treatment-related serious adverse events and no other evidence of harms.

**Interpretation:**

Our findings show that mindfulness-based treatment can be beneficial after non-remission from major depressive disorder following psychological, stepped care treatments. Together with evidence from previous studies of non-remission after pharmacological treatment, our findings establish MBCT, an easily scalable group-based intervention, as a further-line treatment. Implementation of MBCT for patients who continue to have major depressive disorder in routine care settings (NHS Talking Therapies and beyond) is warranted.

**Funding:**

UK National Institute for Health and Care Research Research for Patient Benefit programme.

## Introduction

About half of people with major depressive disorder do not show remission after evidence-based treatment, with symptoms remaining above clinical thresholds, and 20–30% will develop a course in which established treatments repeatedly do not lead to sustained symptom remission.^1^ Recently framed under the heuristic of difficult-to-treat depression,^2^ such disease courses are associated with ongoing functional impairment, reduced quality of life,^3^ and increased morbidity.^4^ People with difficult-to-treat depression show intense use of health-care services, with costs accumulating to high levels as the disorder persists.^5^ Therefore, identification of effective interventions for people who do not show remission after previous treatment is imperative.

Research on further-line treatment has almost exclusively focused on options after pharmacological treatment,^6^ with trials typically finding small-to-moderate effects of additional pharmacological or psychological treatments and diminishing returns with an increasing number of previous treatment attempts.^7^ However, this focus is narrow, given that patients state a preference for psychological treatments.^8^ In England, depression care is primarily organised around a stepped care model of psychological therapies implemented in UK National Health Service (NHS) Talking Therapies services (formerly Improving Access to Psychological Therapies).^9^ Within these services, people whose symptoms do not respond to low-intensity treatment or who present with more complex illness are offered high-intensity treatment, which consists of evidence-based psychotherapies delivered by trained and accredited psychological therapists. In 2023–24, NHS Talking Therapies offered treatment to over 1·83 million people, with those entering treatment receiving a mean of 8·2 sessions.^10^ However, service outcome data show that about 50% of people with depression who complete high-intensity therapies do not reach remission. Only about 10% of these patients qualify for secondary care,^11^ and the remainder are not offered any further specialist care. There has been little research into treatment options when people with depression have not shown remission of symptoms during a previous psychological therapy, and evaluation of the relative benefits and harms of potential approaches has been identified as a research priority by the UK National Institute for Health and Care Excellence (NICE).^12^

**Research in context**

**Evidence before this study**

Establishing the relative benefits and harms of further-line treatments for adults with depression whose symptoms do not show an adequate response to psychological treatment has been identified as a research priority by the UK National Institute for Health and Care Excellence (NICE). This goal is particularly relevant in the context of stepped care approaches, such as UK National Health Service (NHS) Talking Therapies, where people whose symptoms have not shown remission after high-intensity psychological therapy are typically discharged without further specialist input. As a treatment specifically aimed at addressing habitual patterns of maladaptive thinking, mindfulness-based cognitive therapy (MBCT) might be particularly suited for this purpose, and research has already brought preliminary evidence for its use following non-remission after pharmacological treatment. We searched PubMed, Web of Science, Scopus, PsycInfo, and the Cochrane Central Register of Controlled Trials between database inception and Dec 17, 2024, with index and free terms on four tiers: depressive disorders; treatment non-remission or resistance; psychotherapy; and randomised controlled trial (see appendix p 3 for the search string). Among 688 identified individual records, we found no randomised controlled efficacy or effectiveness trial that investigated further-line treatment following symptoms not remitting after psychological therapy. A separate search to establish the state of evidence for MBCT as a further-line treatment regardless of the type of previous treatment identified five studies, four of which had investigated the use of MBCT following symptoms not remitting after pharmacological therapy and one after pharmacological and psychological treatment. Pooled effect sizes indicated a small to moderate post-treatment effect in studies comparing MBCT with treatment as usual (g=–0·40, based on two studies with a total of 156 participants, following removal of one outlier) and a small advantage against other active psychological treatments (g=–0·17, based on three studies with a total of 276 participants). Three of the studies followed up participants over a period of 26 weeks or more, with results providing preliminary evidence that treatment gains from MBCT consolidate over time (appendix pp 3–9). None of these trials included a health economic evaluation.

**Added value of this study**

To our knowledge, this is the first conclusive trial evaluating sustained clinical effectiveness and cost-effectiveness of a further-line treatment for individuals whose symptoms of depression had not shown remission after completing an adequate dose of an evidence-based psychological therapy. Our study shows that MBCT plus usual care is clinically superior and health-economically dominant over usual care alone for individuals whose symptoms have not shown remission after completing the NHS Talking Therapies pathway. Clinical superiority was observed immediately after treatment and sustained at the 34-week primary endpoint. Importantly, MBCT was not only cost-effective, but also potentially cost saving in absolute terms, reducing use of other NHS and public health resource categories relative to treatment as usual.

**Implications of all the available evidence**

Our results show that MBCT can serve as a clinically effective and cost-saving treatment for people with depression whose symptoms have not responded to previous psychological therapy. In combination with previous trials of MBCT following non-remission after pharmacological treatment, our results bring the evidence to a level at which MBCT should be considered for NICE guideline endorsement as a further-line treatment and warrant implementation for this purpose in the UK NHS Talking Therapies service pathway (and by implication in the many health services internationally that follow similar models with comparable populations). More broadly, our findings show that psychological further-line treatment for depression can bring clinical benefit at an affordable price, potentially helping to reduce the long-term disability burden and economic costs associated with difficult-to-treat depression.

A promising candidate to serve as further-line treatment is mindfulness-based cognitive therapy (MBCT),^13^ an 8-week group intervention that uses mindfulness practice as a means of helping patients become better at recognising and disengaging from habitual maladaptive patterns of thinking (referred to as the ability to decentre). This training provides people with skills that remain accessible after the end of the intervention, with evidence suggesting continuing improvements in the ability to decentre as people practise mindfulness after the end of the intervention.^14^ Although originally developed and recommended by guidelines for the prevention of relapse in people with a history of recurrent depression whose symptoms have decreased or subsided, there is preliminary evidence that MBCT can have beneficial effects in people with current depression whose symptoms have not responded to previous pharmacological interventions.^15^, ^16^, ^17^, ^18^ However, more evidence is needed to justify guideline endorsement as a further-line treatment, and no research has investigated the clinical effectiveness and cost-effectiveness of MBCT after non-remission following evidence-based psychological therapy.

We aimed to investigate the clinical effectiveness and cost-effectiveness of adding MBCT, delivered via videoconference, to treatment as usual in reducing symptoms of depression for people who had not shown resolution of symptoms after NHS Talking Therapies high-intensity therapy.

## Methods

### Study design and participants

We conducted a parallel, randomised, controlled, superiority trial to investigate the clinical effectiveness and cost-effectiveness of MBCT plus treatment as usual compared with treatment as usual alone at three sites in the UK (Devon, London, and Sussex). The trial followed a published protocol that is available online and was governed by a trial steering committee, comprising an independent chair, four independent experts, and a patient representative.^18^ The research question was derived from patient consultations and developed with critical lived experience input. A patient advisory group of three experts, led by lived experience team member MR, was involved in all stages of the research (appendix p 65). The research sites recruited patients from 20 NHS Talking Therapies services located across a range of rural and urban areas (appendix p 71). All participating services had recovery rates above 45% and a workforce consisting of at least 40% NHS Talking Therapies therapists, ensuring they were typical and excluding services with outlier characteristics. The trial received ethics approval from the NHS West of Scotland Research Ethics Committee 4 (20/WS/0177) on Jan 18, 2021, and UK Health Research Authority approval on Jan 25, 2021.

Participating services identified people nearing the end of high-intensity treatment who were still showing symptoms at a clinical level or people who, within a 6-month window after the end of high-intensity therapy, showed symptoms at a clinical level without previous remission. Interested patients were contacted for initial screening to confirm caseness and history of at least 12 NHS Talking Therapies high-intensity sessions. NHS Talking Therapies high-intensity therapy consists of structured evidence-based psychological treatment with cognitive behavioural therapy as the primary approach; other evidence-based modalities, such as interpersonal therapy or counselling for depression, are less common but also available. Protocols for moderate to severe depression do not include formal mindfulness training, meaning it was unlikely for patients to have been exposed to such training in their previous treatment. If patient screening was positive, potential participants were invited to take part in a structured clinical interview via videoconferencing and, if eligible, to complete baseline assessments using web-based questionnaires. Baseline questionnaire assessments took place within a window of 4 weeks before random assignment. Informed consent was taken before the start of the clinical interview by asking participants to sign and return a consent form electronically. Potential participants were included if they met the following criteria: non-remission after NHS Talking Therapies high-intensity treatment for depression (at least 12 sessions) defined as a Patient Health Questionnaire-9 (PHQ-9) score of at least 10 at the end of treatment;^19^ current episode of major depressive disorder as assessed through the Mini International Neuropsychiatric Interview for DSM-5 (MINI 7.0.2)^20^ together with a current PHQ-9 score of at least 10; age 18 years or older; and access to a working internet connection and equipment to participate in videoconferencing. Potential participants were excluded if they were eligible for secondary care services; they presented with a level of risk to self (current plans or recent suicidal actions and lack of preventive factors, as assessed by interview) or others that could not be safely managed in a primary care service context, a history of psychotic symptoms, current mania, alcohol or substance use disorder or dependence within the past 3 months, current post-traumatic stress disorder, obsessive-compulsive disorder, or eating disorder; they had any other clinically significant condition that might have put them at risk, or might have affected the result of the trial or their ability to participate in the trial; or they had an insufficient ability to understand English at a level required for the trial. We did not exclude patients who were currently taking antidepressant medication given the pragmatic nature of the trial and the fact that antidepressants are a recommended treatment for the target group; medication use was documented for statistical analysis. We did not assess for seasonal affective disorder. Gender was assessed via self-report, with participants indicating whether they identified as a man, a woman, another gender, or preferred not to say.

The trial was prospectively registered with ISRCTN, 17755571.

### Randomisation and masking

Individual participants were allocated (1:1) to either MBCT plus treatment as usual or treatment as usual through remote random assignment at the UK Clinical Research Collaboration-registered Exeter Clinical Trials Unit. Minimisation was used to support balance across groups using the following factors: depression severity (PHQ-9 score 10–18 *vs* ≥19), antidepressant use at baseline (yes *vs* no), and recruitment site (Devon *vs* London *vs* Sussex). Use of a validated password website ensured concealment. Participants were informed of their allocation by an unmasked member of the research team. As baseline assessment of participants was done before random assignment, there was no risk of disclosure of treatment allocation to the assessor at the time. Use of remote assessments, initiated through automated email, ruled out any potential effects of assessors on assessments of outcomes at 10-week and 34-week follow-up. Due to the nature of the trial, masking to the intervention was not possible for therapists, participants, and trial administrators. However, statistical analyses were done masked to treatment allocation.

### Procedures

Delivery of MBCT followed the treatment manual with minor adaptations (appendix p 63). The intervention, consisting of an individual orientation and eight weekly group-based sessions, follows a sequence that focuses on cultivating general mindfulness skills during the first half of the intervention, and on guiding participants to use these skills to respond more adaptively to what they find to be difficult emotions over the second half. Participation in four or more sessions was considered a minimal adequate dose. Trained MBCT therapists, supported by an assistant, facilitated the intervention for groups with a target size of 13 patients (minimum eight, maximum 16) using videoconferencing on a secure platform (Zoom). The intervention was delivered in five cohorts between October, 2021, and March, 2023. All therapists had qualifications in line with UK Good Practice Guidelines and a track record of teaching MBCT for at least 5 years (mean 11·58, SD 3·89). Therapists took part in a 1-day workshop to familiarise themselves with modifications and received supervision after the orientation and at each weekly session. Treatment fidelity was assessed using the MBCT Adherence Scale based on checks across all eight sessions, and therapist competency was rated using the Mindfulness-Based Interventions: Teaching Assessment Criteria (MBI:TAC) based on video recordings of at least two sessions.^21^, ^22^

Participants in both groups were asked to continue with their usual care and follow the regimens suggested by their general practitioner. We did not restrict treatment as usual (except to suggest that participants in the MBCT plus treatment as usual group did not engage with other psychotherapy during the MBCT intervention) for ethical reasons and because of the pragmatic aims of the trial. Participants in the treatment as usual only group were invited to an interview after random assignment to highlight the importance of their contribution and prevent so-called resentful demoralisation. Both this interview and the orientation interview for MBCT were done within 2 weeks of random assignment. At the end of the trial, participants in the treatment as usual group were offered recommendations for further treatment based on their diagnostic status.

Participants completed assessments at baseline, and at 10 weeks and 34 weeks after random assignment. Participants were asked to complete the follow-up assessments within a 1-week window and were prompted once per week using phone, text, or email for up to 4 weeks, if they did not respond.

### Outcomes

The primary outcome was depression symptoms measured by PHQ-9 score 34 weeks after randomisation.

Secondary outcomes were PHQ-9 score measured at 10 weeks after randomisation, and other clinical outcomes measured at 10-week and 34-week follow-up. Other clinical outcome measures included the Generalised Anxiety Disorder Questionnaire (GAD-7),^23^ Phobia Scale, and the Work and Social Adjustment Scale (WSAS),^24^ all of which were part of NHS Talking Therapies standard outcome reporting for depression when the study started, along with the Warwick–Edinburgh Mental Wellbeing Scale.^25^ Process measures included the Experiences Questionnaire Decentering Scale^26^ and the short version of the Five Facet Mindfulness Questionnaire.^27^ We also computed a series of dichotomous outcomes, which included reliable improvement (reduction greater than the reliable change index: PHQ-9 ≥6, GAD-7 ≥4, or both), recovery (reaching symptom levels below caseness: PHQ-9 <10 and GAD-7 <8), and reliable recovery (showing reliable improvement and reaching recovery) based on the PHQ-9 and GAD-7 (to align with national NHS Talking Therapies metrics) and based on PHQ-9 only (to align with the wider depression literature). Computation of these indices followed NHS Talking Therapies conventions for outcome reporting. To assess potential harms, we computed rates of any deterioration and reliable deterioration (increase in symptoms greater than the reliable change index) on the PHQ-9 and GAD-7 separately.

For health economic analyses, costs were calculated by collecting service use information using the Adult Service Use Schedule (AD-SUS), a self-report measure used in previous depression trials, modified for use online, to which routine unit costs for the year 2021–22 were applied. Discounting was not necessary as costs were collected within 1 year. The AD-SUS also served to assess whether participants were taking medication for mental health problems, including depression, anxiety, psychosis, or sleep problems. We collected data on all service use, not just use related to mental health conditions, because there is evidence that successful treatment in NHS Talking Therapies can reduce use of all health-care and public services.^28^ Data on the use of the MBCT intervention were collected via therapist records and costs estimated using the standard approach set out by Curtis,^29^ acknowledging the challenges of costing group-based interventions. Outcomes for the economic evaluation were quality-adjusted life-years (QALYs), calculated using health utilities derived from the EQ-5D-5L according to the latest guidance from NICE.^30^

To identify risk issues, research assistants screened all questionnaires within 72 h of completion. In addition to the trial assessments, participants in the MBCT plus treatment as usual group provided weekly PHQ-9 ratings during the intervention. Risk assessments were done after reliable deterioration from the previous assessment or week or an increase of 1 point or more on the suicidal ideation question. An independent clinician assessed all serious adverse events for potential trial or intervention relatedness.

### Choice of primary outcome measure

The PHQ-9 is a nine-item self-report measure of depression severity (range from 0, no symptoms, to 27, severely depressed, with scores ≥10 indicating caseness; reliable improvement is a 6-point reduction), which represents an integral part of the management of depression in the NHS Talking Therapies pathway. The PHQ-9 aligns with DSM-5 criteria for depression, has excellent sensitivity and specificity,^19^ and is recommended as a core depression measure by the Common Measures in Mental Health Initiative. The PHQ-9 is widely used in research, is free, and has been translated into multiple languages.

### Statistical analysis

We aimed to recruit a total sample of 234 participants (117 in each group, 78 per site) to have 90% power for detecting a minimal clinically important difference of 2·59 on the PHQ-9, with a two-sided threshold of 0·05, assuming an SD of 5·4, and allowing for 20% attrition. This minimal clinically important difference was conservatively chosen to be at the smaller end of previously tested reliability scenarios.^31^

Statistical analyses were specified in a statistical analysis plan that was signed off before the end of the trial on July 14, 2023 (appendix pp 10–27). The primary and all secondary outcomes were analysed according to the intention-to-treat principle (all participants were included in the analysis according to their random allocation, irrespective of the treatment received) including observed data only. Continuous outcomes were analysed using linear regression models. Binary outcomes were analysed using logistic regression. All analyses adjusted for participant covariates used in random assignment (depression severity, antidepressant use at baseline, and recruitment site), with adjustment for baseline scores for continuous outcomes. Analyses of the continuous PHQ-9 outcomes adjusted for baseline PHQ-9 but not depression severity due to collinearity. Primary analyses included all data collected within the overall window for each follow-up time.

A series of sensitivity analyses were done to test robustness of findings. To investigate the clustering effect of NHS Talking Therapies services, mixed effects regression models were performed with a random effect on NHS Talking Therapies service. To investigate effects under conditions of different inclusion criteria, a sensitivity analysis examined differences between treatment groups only for participants who did not show remission and who did not show reliable improvement on the PHQ-9 in their high-intensity therapy. The effect of time of assessment was investigated by excluding any data collected outside the initial 7 days of the assessment window (primary outcome only). We also did a complier average causal effect analysis for continuous outcomes only, to estimate the treatment effect while accounting for non-adherence to treatment. A participant in the intervention arm was considered to have complied if a minimum of four sessions were attended, which is standard practice for MBCT trials.

To address the potential effects of missing data, further sensitivity analyses used multiple imputation to impute missing outcome data for the primary outcome and all secondary continuous outcomes at 34 weeks. Multiple imputation using chained equations was applied; the imputation algorithm included age and age of onset (as these baseline characteristics were found to be predictive of missingness of the primary outcome) and outcome data reported at 10-week follow-up, as well as treatment group and minimisation variables. As a further analytical strategy to address missing data, a repeated measures analysis was done, using a mixed effects linear regression model with a random effect on participant and including an interaction term between treatment group and timepoint. Clinical outcome analyses were done using Stata version 17.1 and later.

Health economic analyses were specified in a health economic analysis plan that was signed off before the end of the trial (appendix pp 28–34). The primary economic evaluation took a health and social care perspective, including costs to primary, secondary, and tertiary health and social care services, as required for evidence presented to UK decision maker NICE. A full list of included services is in the appendix (p 49). Additionally, the cost perspective was broadened to include the costs of self-reported time off productivity losses in a secondary evaluation, since these are known to be relevant and important in those attending NHS Talking Therapies services. Costs were expressed in pounds sterling (£) in 2021–22 prices. Intention-to-treat analyses using observed data and analyses based on imputed data were done. Multiple imputation methods were used based on the assumption that data were missing at random. Differences in overall mean costs and overall mean QALYs between the study groups across 34 weeks were analysed using a regression model adjusting for depression severity (PHQ-9 <19 *vs* ≥19), antidepressant use at baseline, and recruitment site with the addition of baseline costs. Non-parametric bootstrapping of trial data was used to determine the level of sampling uncertainty surrounding the costs and effectiveness estimates and summary cost-effectiveness measures.^32^ Cost-effectiveness acceptability curves were generated to consider the probability of MBCT being cost-effective for willingness to pay values between £0 and £50 000.

An independent data monitoring committee, consisting of an independent chair, independent statistician, and independent clinician, monitored progress, data quality, and safety of the trial.

### Role of the funding source

The funder of the study had no role in study design, data collection, data analysis, data interpretation, or writing of the report.

## Results

**Figure 1:**
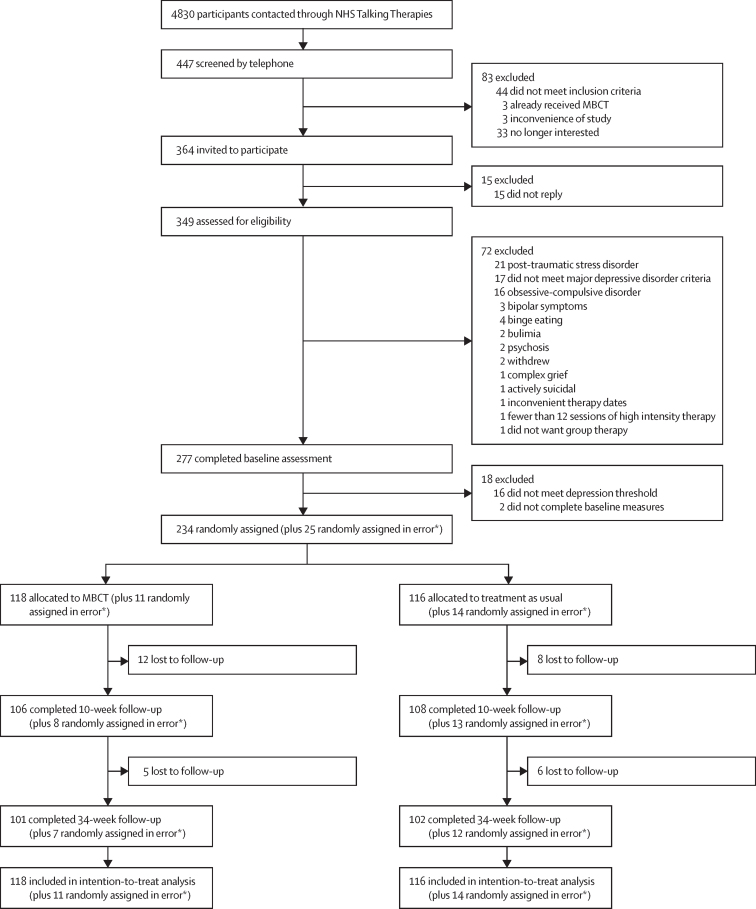
Trial profile MBCT=mindfulness-based cognitive therapy. NHS=UK National Health Service. *When the research team retrospectively checked inclusion criteria against NHS Talking Therapies service records from Nov 22, 2022, to Jan 23, 2023, it became obvious that patient self-reports during assessments had been inconsistent with records in 25 cases. For the remaining recruitment of the trial, service records were checked before random assignment, assuring that for all of the 234 eligible and randomly assigned participants, service records were consistent with inclusion criteria of the trial.

Between April 20, 2021, and Jan 24, 2023, 349 patients were assessed for eligibility through clinical assessment, 277 of whom met initial criteria and were invited to complete baseline assessments (figure 1). 259 patients were initially found to be eligible and randomly assigned. However, 25 patients were deemed to be randomly assigned in error after retrospective checks of self-reported inclusion criteria against NHS Talking Therapies service records indicated discrepancies (in 23 cases the last PHQ-9 score recorded for the NHS Talking Therapies high-intensity treatment of the patients did not meet the criterion of being above the threshold for caseness of PHQ-9 ≥10, in one case the number of treatment sessions was recorded as below 12, and in one case the last recorded PHQ-9 score did not meet the criterion of caseness and the number of recorded high-intensity sessions was below 12). After discussion with the trial steering committee and independent data monitoring committee, it was agreed to exclude these patients from primary analyses. However, as they met all other eligibility criteria, including a PHQ-9 score above caseness at baseline assessment, it was agreed to include these participants in a sensitivity analysis for the primary outcome only. Of the 234 eligible and correctly randomly assigned participants, 118 were assigned to MBCT plus treatment as usual and 116 to treatment as usual. 19 (16%) of 118 participants allocated to MBCT either did not start the intervention (nine [7%] participants) or dropped out of treatment before receiving the minimum dose of four sessions (ten [8%] participants). Outcome data were available for 214 (91%) of 234 participants at 10-week follow-up and 203 (87%) participants at 34-week follow-up (figure 1). The final follow-up closed on Oct 17, 2023.

166 (71%) of 234 eligible participants identified as women, 65 (28%) as men, one (<1%) as other, and two (1%) preferred not to say (table 1). The mean participant age was 42·5 years (SD 13·9). 201 (86%) of 234 participants were White. Participants had experienced a mean of 6·08 previous episodes of depression (SD 13·81) before the current episode. The mean age of onset was in young adulthood (mean 20·30 years, SD 10·47). More than a third of participants reported low or moderate levels of suicidality and about two-thirds had at least one comorbid anxiety disorder at entry into the study (table 1). In terms of previous NHS Talking Therapies high-intensity therapy, participants had received a mean of 17·4 sessions (SD 4·67, range 12–40) and shown a mean improvement of 2·71 (SD 5·20) on the PHQ-9.

**Table 1:** Baseline demographics

		**MBCT plus treatment as usual (n=118)**	**Treatment as usual (n=116)**	**Total sample (n=234)**
Age, years		42·9 (13·3),[19–75]	42·0 (14·4), [18–81]	42·5 (13·9), [18–81]
Gender				
	Woman	82 (69%)	84 (72%)	166 (71%)
	Man	34 (29%)	31 (27%)	65 (28%)
	Other	0	1 (1%)	1 (<1%)
	Prefer not to say	2 (2%)	0	2 (1%)
Marital status				
	Single	37 (31%)	49 (42%)	86 (37%)
	Married or civil partnership	34 (29%)	27 (23%)	61 (26%)
	Cohabiting	13 (11%)	6 (5%)	19 (8%)
	Separated or divorced	13 (11%)	14 (12%)	27 (12%)
	Widowed	3 (3%)	4 (3%)	7 (3%)
	In a long-term relationship	16 (14%)	14 (12%)	30 (13%)
	Prefer not to say	2 (2%)	2 (2%)	4 (2%)
Ethnicity				
	Asian or Asian British	2 (2%)	5 (4%)	7 (3%)
	Black, African, Caribbean or Black British	3 (3%)	3 (3%)	6 (3%)
	Mixed or multiple ethnic groups	7 (6%)	5 (4%)	12 (5%)
	Other	1 (1%)	4 (3%)	5 (2%)
	White	103 (87%)	98 (84%)	201 (86%)
	Prefer not to say	2 (2%)	1 (1%)	3 (1%)
Highest educational attainment				
	None	2 (2%)	3 (3%)	5 (2%)
	GCSE* or equivalent	19 (16%)	15 (13%)	34 (15%)
	A-Level† or equivalent	29 (25%)	26 (22%)	55 (24%)
	Undergraduate or equivalent	34 (29%)	33 (28%)	67 (29%)
	Postgraduate or equivalent	33 (28%)	36 (31%)	69 (29%)
	Prefer not to say	1 (1%)	3 (3%)	4 (2%)
Annual household income, £				
	0–10 000	19 (16%)	7 (6%)	26 (11%)
	10 001–20 000	16 (14%)	22 (19%)	38 (16%)
	20 001–30 000	14 (12%)	24 (21%)	38 (16%)
	30 001–40 000	15 (13%)	12 (10%)	27 (12%)
	40 001–50 000	12 (10%)	8 (7%)	20 (9%)
	50 001–60 000	12 (10%)	8 (7%)	20 (9%)
	60 001–70 000	5 (4%)	2 (2%)	7 (3%)
	70 001–80 000	3 (3%)	3 (3%)	6 (3%)
	80 001–90 000	4 (3%)	1 (1%)	5 (2%)
	90 001–100 000	0	2 (2%)	2 (1%)
	100 001–150 000	1 (1%)	5 (4%)	6 (3%)
	150 001–200 000	1 (1%)	0	1 (<1%)
	≥200 001	0	0	0
	Prefer not to say	16 (14%)	22 (19%)	38 (16%)
Previous episodes of depression		5·43 (10·13),[1–80]	6·72 (16·68), [1–100]	6·08 (13·81), [1–100]
Age of onset, years		20·69 (10·57), [5–52]	19·92 (10·40), [3–73]	20·30 (10·47), [3–73]
Currently taking antidepressant medication		69 (58%)	70 (60%)	139 (59%)
Suicidality (Mini International Neuropsychiatric Interview)				
	None	78 (66%)	68 (59%)	146 (62%)
	Low	29 (25%)	37 (32%)	66 (28%)
	Moderate	11 (9%)	11 (9%)	22 (9%)
Comorbid anxiety disorder				
	Panic disorder	21 (18%)	26 (22%)	47 (20%)
	Agoraphobia	38 (32%)	28 (24%)	66 (28%)
	Social anxiety disorder	32 (27%)	28 (24%)	60 (26%)
	Generalised anxiety disorder	65 (55%)	50 (43%)	115 (49%)
	Any anxiety disorder	73 (62%)	75 (65%)	148 (63%)
Bipolar history				
	Past manic episode	21 (17%)	26 (22%)	47 (20%)
	Past hypomanic episode	38 (32%)	28 (24%)	66 (28%)

Data are mean (SD), [range] or n (%). Participants randomly assigned in error (n=25) are not included in the table; sociodemographic and clinical characteristics of these participants did not differ from those included in the trial. MBCT=mindfulness-based cognitive therapy.

* GCSEs are academic qualifications in the UK typically taken by students aged 14–16 years at the end of secondary school (year 10 and year 11), equivalent to the US High School Diploma.

† A-Levels are typically taken by students aged 16–18 years after completing GCSEs, equivalent to Advanced Placement in the USA or the International Baccalaureate Diploma.

Treatment fidelity as assessed on the MBCT Adherence Scale was excellent (mean 31·25, SD 1·86, on a scale ranging from 0 to 34). Ratings of therapist competence on the MBI:TAC were at or above the competent level for all groups. Rates of attendance at the eight weekly group sessions ranged from 106 (90%) of 118 participants at the first session to 81 (69%) of 118 at the seventh session, with 99 (84%) of the 118 MBCT participants receiving at least the minimal adequate dose of four group sessions. Average self-reported weekly engagement in home practice (assessed using electronic versions of the MBCT practice records) ranged from 53% to 77% for formal practices (guided meditations and movement practices) and 35–72% for informal practices (shorter practices to bring mindfulness into daily life), the mean number of formal practices was 18·07 (SD 13·29), and the mean number of informal practices was 38·57 (31·57). Engagement in practice was not significantly correlated with PHQ-9 scores at entry (p=0·52 for formal practice, p=0·45 for informal practice). Inspection of resource use over the 34-week trial period indicated that most participants were taking antidepressant medication (65 [69%] of 94 participants in the MBCT plus treatment as usual group and 71 [74%] of 95 participants in the treatment as usual group) and a sizeable number of participants engaged with psychological non-trial treatment (25 [27%] of 94 participants in the MBCT plus treatment as usual group and 38 [40%] of 95 participants in the treatment as usual group). There was little engagement with psychiatrists, community psychiatry nursing, key workers, home treatment teams, or arts-related therapies (all <6%; appendix p 50), suggesting that treatment as usual was relatively homogeneous and broadly in line with NICE recommendations, which suggest antidepressants, switching to another psychological therapy, or combined treatment.

The primary intention-to-treat analysis indicated that MBCT plus treatment as usual was superior to treatment as usual alone in reducing symptoms of depression at 34-week follow-up, with an adjusted difference on the PHQ-9 of –2·49 (95% CI –3·89 to –1·09; p=0·0006; table 2), which translates into an effect size of Cohen's d –0·41 (95% CI –0·67 to –0·15). Analyses of continuous secondary outcomes at 10-week and 34-week follow-ups showed that MBCT plus treatment as usual was superior across all measures apart from the WSAS at 10-week follow-up and the Phobia Scale (table 2).

**Table 2:** Summary of linear models predicting continuous outcomes at 10-week and 34-week follow-up (intention-to-treat analysis, observed data)

		**Baseline**		**10-week follow-up**		**34-week follow-up**		Between-group differences (95% CI)*		p value*	
		n	Mean (SD)	n	Mean (SD)	n	Mean (SD)	10-week follow-up	34-week follow-up	10-week follow-up	34-week follow-up
PHQ-9											
	MBCT plus treatment as usual	118	17·95 (3·92)	106	13·94 (5·22)	101	12·64 (5·45)	−2·50 (−3·64 to −1·35)	−2·49 (−3·89 to −1·09)	<0·0001	0·0006
	Treatment as usual	116	17·77 (3·83)	108	16·10 (4·77)	102	14·88 (5·58)	..	..	..	..
GAD-7											
	MBCT plus treatment as usual	118	13·48 (4·64)	106	11·11 (5·00)	101	10·49 (5·24)	−2·06 (−3·06 to −1·06)	−1·72 (−2·95 to −0·48)	<0·0001	0·0064
	Treatment as usual	116	13·22 (4·63)	108	12·78 (4·53)	102	11·96 (5·18)	..	..	..	..
Phobia Scale											
	MBCT plus treatment as usual	118	10·52 (6·35)	106	9·99 (6·40)	101	9·37 (6·49)	−0·09 (−0·98 to 0·80)	−0·60 (−1·61 to 0·40)	0·84	0·24
	Treatment as usual	116	11·03 (6·50)	108	10·76 (6·22)	102	10·48 (6·47)	..	..	..	..
WSAS											
	MBCT plus treatment as usual	118	23·56 (6·39)	106	21·73 (8·23)	101	19·82 (8·48)	−1·14 (−3·07 to 0·09)	−1·95 (−3·82 to −0·09)	0·065	0·040
	Treatment as usual	116	23·95 (7·47)	108	23·85 (7·95)	102	22·18 (8·80)	..	..	..	..
WEMWBS											
	MBCT plus treatment as usual	118	30·82 (5·84)	106	35·58 (7·64)	101	36·58 (7·97)	3·32 (1·77 to 4·86)	3·24 (1·35 to 5·13)	<0·0001	0·0009
	Treatment as usual	116	31·29 (5·68)	108	32·58 (6·72)	102	33·20 (7·49)	..	..	..	..
EQ Dec											
	MBCT plus treatment as usual	118	27·24 (5·54)	106	31·24 (5·89)	101	31·18 (6·83)	3·46 (2·22 to 4·70)	3·07 (1·60 to 4·54)	<0·0001	<0·0001
	Treatment as usual	116	27·56 (5·11)	108	27·91 (5·03)	102	28·15 (5·37)	..	..	..	..
FFMQ											
	MBCT plus treatment as usual	118	38·25 (7·06)	106	43·41 (7·16)	101	43·39 (8·77)	5·92 (4·47 to 7·37)	4·61 (2·70 to 6·51)	<0·0001	<0·0001
	Treatment as usual	116	39·05 (7·13)	108	38·09 (6·89)	102	38·98 (7·50)	..	..	..	..

Higher scores on the PHQ-9, GAD-7, Phobia Scale, and WSAS indicate higher levels of symptoms or worse functioning; higher scores on the WEMWBS, EQ Dec, and FFMQ indicate better psychological functioning. EQ Dec=Experiences Questionnaire Decentering Scale. FFMQ=Five Facet Mindfulness Questionnaire. GAD-7=Generalised Anxiety Disorder-7 scale. MBCT=mindfulness-based cognitive therapy. PHQ-9=Patient Health Questionnaire-9. WEMWBS=Warwick-Edinburgh Mental Well-Being Scale. WSAS=Work and Social Adjustment Scale.

* Linear models predicting continuous outcomes were adjusted for depression severity (PHQ-9 score <19 *vs* ≥19), antidepressant use at baseline, recruitment site, and baseline score, apart from linear models for PHQ-9, where dichotomised depression severity was not included due to collinearity with the PHQ-9 baseline score.

Logistic regression analyses of dichotomous secondary outcomes indicated that significantly more patients in the MBCT plus treatment as usual group than the treatment as usual alone group reached recovery, reliable improvement, and reliable recovery based on the PHQ-9 at both 10 weeks and 34 weeks (table 3). Joint rates of recovery, reliable improvement, and reliable recovery on the PHQ-9 and GAD-7, the official NHS Talking Therapies metric, were significant at 10-week follow-up but did not reach significance for recovery and reliable recovery rates at 34-week follow-up. Absolute rates of recovery at 34 weeks based on the PHQ-9 were 27% in the MBCT plus treatment as usual group and 15% in the treatment as usual alone group (table 3).

**Table 3:** Summary of logistic regression models predicting dichotomous outcomes at 10-week and 34-week follow-up

		**10-week follow-up**	**34-week follow-up**	Odds ratio (95% CI)*		p value*	
				10-week follow-up	34-week follow-up	10-week follow-up	34-week follow-up
**PHQ-9 only**							
Recovery†		..	..	2·29 (1·03–5·09)	2·12 (1·11–4·06)	0·042	0·023
	MBCT plus treatment as usual	22/118 (19%)	33/118 (28%)	..	..	..	..
	Treatment as usual	11/116 (9%)	18/116 (16%)	..	..	..	..
Reliable improvement†		..	..	3·05 (1·56–5·95)	1·84 (1·04–3·26)	0·0011	0·036
	MBCT plus treatment as usual	37/118 (31%)	46/118 (39%)	..	..	..	..
	Treatment as usual	16/116 (14%)	31/116 (27%)	..	..	..	..
Reliable recovery†		..	..	3·72 (1·41–9·84)	2·36 (1·15–4·84)	0·0080	0·020
	MBCT plus treatment as usual	19/118 (16%)	27/118 (23%)	..	..	..	..
	Treatment as usual	6/116 (5%)	13/116 (11%)	..	..	..	..
Deterioration†		..	..	0·37 (0·19–0·73)	0·15 (0·06–0·39)	0·0038	0·0001
	MBCT plus treatment as usual	17/118 (14%)	6/118 (5%)	..	..	..	..
	Treatment as usual	35/116 (30%)	29/116 (25%)	..	..	..	..
Reliable deterioration†		..	..	0·37 (0·03–4·54)	1·00 (0·13–7·43)	0·44	1·00
	MBCT plus treatment as usual	1/118 (1%)	2/118 (2%)	..	..	..	..
	Treatment as usual	2/116 (2%)	2/116 (2%)	..	..	..	..
**GAD-7 only**							
Deterioration‡		..	..	0·40 (0·22–0·74)	0·55 (0·31–1·01)	0·0037	0·054
	MBCT plus treatment as usual	21/118 (18%)	25/118 (21%)	..	..	..	..
	Treatment as usual	40/116 (34%)	38/116 (33%)	..	..	..	..
Reliable deterioration‡		..	..	0·77 (0·30–1·95)	0·96 (0·40–2·28)	0·58	0·93
	MBCT plus treatment as usual	9/118 (8%)	12/118 (10%)	..	..	..	..
	Treatment as usual	11/116 (9%)	13/116 (11%)	..	..	..	..
**PHQ-9 and GAD-7**							
Recovery§		..	..	4·69 (1·45–15·14)	1·85 (0·86–3·98)	0·010	0·11
	MBCT plus treatment as usual	15/118 (13%)	21/118 (18%)	..	..	..	..
	Treatment as usual	4/116 (3%)	12/116 (10%)	..	..	..	..
Reliable improvement§		..	..	2·69 (1·51–4·81)	2·00 (1·17–3·43)	0·0008	0·012
	MBCT plus treatment as usual	50/118 (42%)	58/118 (49%)	..	..	..	..
	Treatment as usual	26/116 (22%)	39/116 (34%)	..	..	..	..
Reliable recovery§		..	..	8·97 (1·94–41·57)	2·27 (0·98–5·26)	0·0050	0·056
	MBCT plus treatment as usual	14/118 (12%)	19/188 (16%)	..	..	..	..
	Treatment as usual	2/116 (2%)	9/116 (8%)	..	..	..	..
Reliable deterioration§		..	..	0·59 (0·23–1·54)	1·16 (0·48–2·82)	0·28	0·75
	MBCT plus treatment as usual	8/118 (7%)	12/118 (10%)	..	..	..	..
	Treatment as usual	12/116 (10%)	11/116 (9%)	..	..	..	..

Data are n/N (%), unless otherwise indicated. Denominators are the full number of participants entering the treatment group at random assignment to align with the so-called demonstrated recovery principle used for UK National Health Service Talking Therapies statistics, which posits that unless questionnaire data explicitly show recovery, patients should be counted as not having reached remission, including those for whom no further data are available. Not all participants reported anxiety levels at the level of caseness at baseline. GAD-7=Generalised Anxiety Disorder-7 scale. MBCT=mindfulness-based cognitive therapy. PHQ-9=Patient Health Questionnaire-9.

* Logistic regression models were adjusted for depression severity (PHQ-9 score <19 *vs* ≥19), antidepressant use at baseline, and recruitment site.

† Recovery is reduction of depression symptoms to a level below caseness (PHQ-9 <10), reliable improvement is reduction in depression symptom score greater than the reliable change index (PHQ-9 ≥6), reliable recovery is reduction of depression symptoms to reach reliable improvement and recovery, deterioration is any increase in depression symptom score on the PHQ-9, and reliable deterioration is an increase in depression symptom score greater than the reliable change index (PHQ-9 ≥6).

‡ Deterioration is any increase in anxiety symptom score on the GAD-7 and reliable deterioration is an increase in anxiety symptom score greater than the reliable change index (GAD-7 ≥4).

§ Recovery is reduction of depression and anxiety symptoms to a level below caseness (PHQ-9 <10 and GAD-7 <8), reliable improvement is reduction in depression symptom score and anxiety symptom score greater than reliable change index (PHQ-9 ≥6 or GAD-7 ≥4) or reduction in depression symptom score or anxiety symptom score without reliable deterioration (PHQ-9 ≥6, GAD-7≥4) in the other, and reliable recovery is reliable improvement and recovery.

We found no evidence of trial-related or treatment-related harms. Reliable deteriorations between assessment timepoints in depressive symptoms were rare in both groups, and reliable deteriorations in anxiety symptoms occurred in about 10% of participants in both groups (table 3). Seven serious adverse events were recorded over the duration of the trial, two in participants in the MBCT plus treatment as usual group, three in participants in the treatment as usual group, and two in participants who had not been randomly assigned. Events included hospital stays for weight loss surgery, gallbladder removal, burn, infections, heart surgery, and suspected sepsis, as well as a disclosure of suicidality before trial screening. Three events occurred in women, three in men, and one in a participant who preferred not to report their gender. All events were deemed unrelated to the trial and intervention by the independent clinical monitor (appendix p 69).

**Figure 2:**
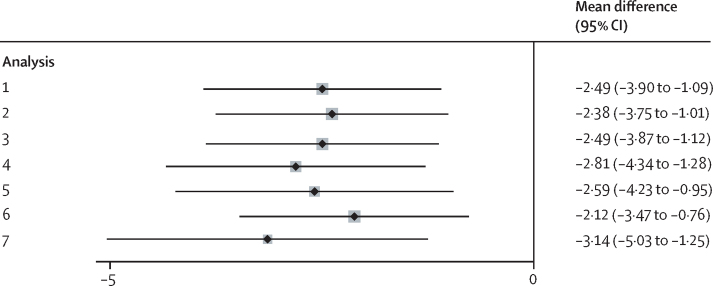
Sensitivity analyses for the primary outcome (PHQ-9 at 34-week follow-up) Point estimates for mean difference and 95% CI. Analysis 1 is intention to treat, observed data only. Analysis 2 is observed and imputed data. Analysis 3 is including random effect on NHS Talking Therapies service. Analysis 4 is complier average causal effect analysis. Analysis 5 is excluding participants with reliable improvement on PHQ-9 during initial NHS Talking Therapies treatment. Analysis 6 is including all randomly assigned participants. Analysis 7 is excluding responses out of window. NHS=UK National Health Service. PHQ-9=Patient Health Questionnaire-9.

Sensitivity analyses showed that the pattern of results remained unchanged when considering the effect of NHS Talking Therapies services, when considering the effect of individual therapist or therapist seniority, when including only patients who did not reach symptom levels below caseness and had shown no reliable improvement (84 of 118 participants in the MBCT plus treatment as usual group and 79 of 116 participants in the treatment as usual alone group), and when excluding any data collected outside the initial 7 days of the assessment window as well as when accounting for non-compliance using complier average causal effect analysis. The pattern of results also remained unchanged when analyses were done on imputed data and when we did a repeated measures analysis including participants with follow-up data reported for at least one follow-up time. Mean differences on the primary outcome across the different sensitivity analyses are shown in figure 2 (appendix pp 35–48).

**Figure 3:**
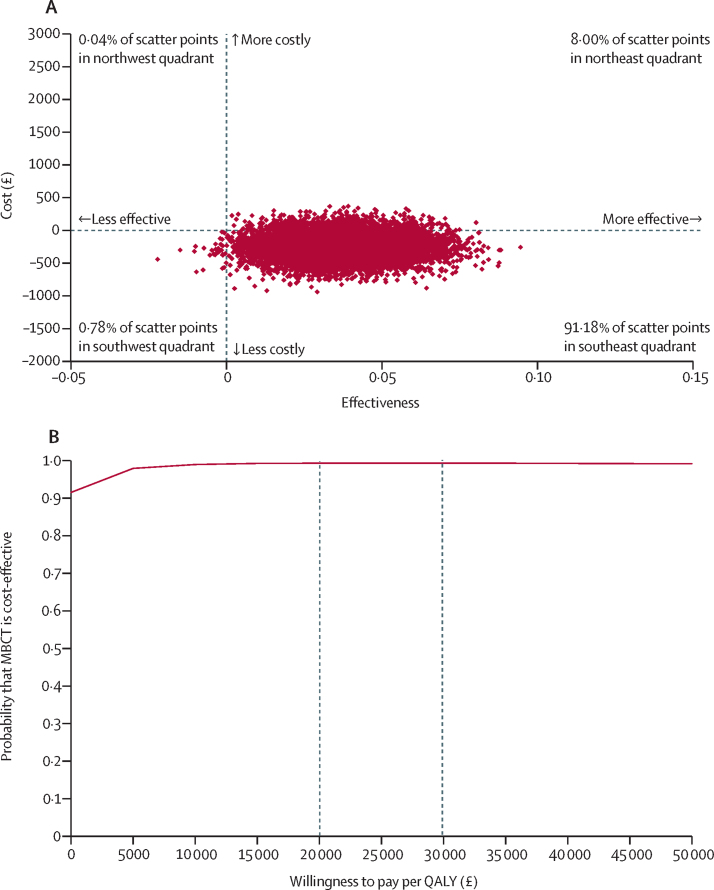
Bootstrapped mean differences in costs and QALYs at 34-week follow-up (health and social care perspective, complete case; (A) and cost-effectiveness acceptability curve showing the probability that MBCT is cost-effective compared with treatment as usual at different values of willingness to pay thresholds per QALY at 34-week follow-up (health and social care perspective, complete case; (B) Estimates of cost-effectiveness ratios based on 10 000 bootstrapped replications of a model adjusted for depression severity (Patient Health Questionnaire-9 score <19 *vs* ≥19), antidepressant use at baseline, recruitment site, and baseline costs. MBCT=mindfulness-based cognitive therapy. QALYs=quality-adjusted life-years.

The cost of the MBCT intervention was estimated to be £10·12 per person per session, assuming a group size of 13. Over the 34 weeks of the study, health and social care costs were lower and utilities scores higher in the MBCT plus treatment as usual group (mean costs unadjusted difference –£316·09, adjusted difference –£245·23, 95% CI –581·92 to 91·46; p=0·15; utility scores unadjusted difference 0·064, adjusted difference 0·564, 95% CI –0·004 to 0·117; p=0·069). Non-parametric bootstrapping of mean differences in costs and QALYs at 34-week follow-up resulted in 91% of scatter points situated in the cost-effectiveness plane quadrant, indicating higher effectiveness and less cost for MBCT plus treatment as usual (figure 3). The cost-effectiveness acceptability curve for the primary health and social care perspective indicated a 95% probability for MBCT plus treatment as usual being cost-effective at a willingness to pay level of less than £5000 and had an estimated 99% chance of being cost-effective at the £20 000–30 000 threshold preferred by NICE. Secondary analyses considering the widened health and social care and productivity perspective brought similar results, although effects were numerically smaller (appendix pp 59–62). Cost-effectiveness analyses using the NHS Talking Therapies reliable recovery outcome showed a similar pattern and are detailed in the appendix (pp 53, 56–57). The pattern of results remained unchanged when analyses were repeated with missing data imputed via multiple imputation (appendix pp 58, 61).

## Discussion

We found MBCT plus treatment as usual, delivered via videoconference, to be superior to treatment as usual alone in reducing depressive symptomatology in people whose symptoms had not reached remission after NHS Talking Therapies high-intensity therapy, with effects of small to moderate size maintained up to 6 months after the end of treatment. Evaluation from a health and social care perspective indicated that MBCT was economically dominant, meaning that its introduction would not only increase clinical effectiveness but also reduce health and social care costs compared with treatment as usual, even when considering the additional resources required to provide the treatment.

The current sample was characterised by complex courses with early onset, high numbers of recurrence, and considerable comorbidity. These factors, plus the fact that about 70% of those who provided data at 34 weeks had also been taking antidepressant medication, suggest that non-remission after NHS Talking Therapies high-intensity therapy was unlikely to be the first failed treatment attempt and confirms that this group shows many characteristics of difficult-to-treat depression. Previous research on further-line treatments, mostly pharmacological, has found diminishing benefit from each additional treatment introduced, with reanalyses of the STAR*D trial suggesting remission rates between 8% and 19% from first to second treatment step and 2% and 6% from second to third treatment step.^7^ The conservatively estimated between-group difference in sustained remission of 12% in our study is similar to these rates and has the potential to meaningfully reduce disease burden. The small to medium effect size advantage of MBCT is also similar to findings from previous trials of psychological treatment in people with chronic or treatment-resistant depression, with an estimated general effect size of 0·42.^33^ It is unlikely that the benefits we observed for MBCT would emerge from any psychological treatment irrespective of treatment type, given that previous trials of long-term psychodynamic therapy and interpersonal psychotherapy for treatment-resistant depression found no significant advantage of these treatments compared with treatment as usual.^34^, ^35^

In addition to reducing depressive symptomatology, MBCT plus treatment as usual was superior to treatment as usual alone in reducing symptoms of generalised anxiety and increasing mental wellbeing more broadly. These findings are in line with the view that mindfulness-based interventions can target transdiagnostically relevant processes, although effects on anxiety appeared more limited than effects on depression, as reflected by the fact that rates of recovery and reliable recovery based on the combination of PHQ-9 and GAD-7 did not reach significance at 34 weeks. Post-treatment gains were maintained and skills consolidated through the follow-up, indicating that patients continued to benefit beyond the intervention, which is consistent with the intention of MBCT to provide patients with portable tools that help them stay well.

When considering the ratio of costs and effects, we found that MBCT plus treatment as usual was dominant over treatment as usual alone and that the certainty of cost-effectiveness approached ceiling levels far below the conventional threshold of £20 000 per QALY used by NICE. These findings offer an unusually strong indication of cost-effectiveness from a health and social care cost perspective, arising in the context of considerable and continuing burden associated with difficult-to-treat depression and despite only small to moderate clinical effects. As such, our findings argue for the introduction of MBCT for people whose symptoms have not shown remission in NHS Talking Therapies and highlight the potential health economic benefits of extending systematic care for this group more widely.

Confidence in our findings can be high given that rates of dropout from the trial were small and definition of remission was rigorous. It is unlikely that previous non-remission was due to misdiagnosis or failure to implement evidence-based interventions correctly given the standard procedures in place in NHS Talking Therapies services. By requiring participants to have received at least 12 sessions of high-intensity therapy, the study protected against inclusion of patients whose symptoms might not have reached remission due to insufficient dosage, and results remained unchanged in a sensitivity analysis based on patients who had not reached remission and not shown any reliable improvement in their previous therapy.

A limitation of the trial is the relatively restricted follow-up period of 6 months. Furthermore, a preponderance of female and White participants limits representativeness. We did not include gender-specific analyses as the trial was not powered for subgroup analyses. There was little information on the type and quality of psychological treatment patients received as treatment as usual.

This study adds robust evidence to existing research that has brought encouraging preliminary support for the use of MBCT in patients whose symptoms of depression have not responded to pharmacological therapies. It brings combined evidence to a level at which MBCT should be considered for guideline endorsement as a further-line treatment in the UK (appendix p 3). In conclusion, MBCT should be offered as an option to patients whose symptoms have not shown remission after evidence-based therapies for depression, and efforts are needed to make this treatment more widely available.

### Contributors

### Data sharing

Data sharing will be enabled using a controlled access model in line with Good Practice Principles for Sharing Individual Participant Data from Publicly Funded Clinical Trials from the UK Medical Research Council. Scientists seeking to access the data for use in future projects must do so via request to the corresponding author (TB) and projects using the data must have been approved in accordance with contemporary UK ethical and regulatory processes pertaining to the release of anonymised data under these circumstances. We will follow recommendations on anonymising and curating trial data for sharing. Trial data will be stored in repositories at the site of the study sponsor (Sussex Partnership NHS Foundation Trust) and at the University of Exeter.

## Declaration of interests

TB is the author of a book on mindfulness-based cognitive therapy (MBCT). TB, CS, and FAR regularly provide workshops on mindfulness-based interventions. CS is a member of a training organisation that has been commissioned by NHS England to deliver MBCT training across NHS Talking Therapies services in England and co-leads the Sussex Mindfulness Centre. TB and CS are co-investigators of a programme grant evaluating an adapted MBCT course for adolescents experiencing depression. CS is co-investigator on a grant evaluating an adapted MBCT course for NHS staff. BDD is the lead of the University of Exeter AccEPT clinic, which offers courses of MBCT. AHY serves as principal investigator for several psychopharmacological trials for treatment-resistant depression and has provided paid lectures and participated on advisory boards for Flow Neuroscience, Novartis, Roche, Janssen, Takeda, Noema Pharma, Compass, Astrazenaca, Boehringer Ingelheim, Eli Lilly, LivaNova, Lundbeck, Sunovion, Servier, Janssen, Allegan, Bionomics, Sumitomo Dainippon Pharma, Sage, and Neurocentrx. All other authors declare no competing interests.
