# Supplementary material for: Mindfulness-based cognitive therapy versus treatment as usual after non-remission with NHS Talking Therapies high-intensity psychological therapy for depression: a UK-based clinical effectiveness and cost-effectiveness randomised, controlled, superiority trial
Source: Lancet Psychiatry. 2025 Jun;12(6):433–46. doi: 10.1016/S2215-0366(25)00105-1 (PMC12078190; doi:10.1016/S2215-0366(25)00105-1)
Supplement: Supplementary appendix [file mmc1.pdf]

THE LANCET
Psychiatry

Supplementary appendix

This appendix formed part of the original submission and has been peer reviewed. We post it as supplied by the authors.

Supplement to: Barnhofer T, Dunn BD, Strauss C, et al. Mindfulness-based cognitive therapy versus treatment as usual after non-remission with NHS Talking Therapies high-intensity psychological therapy for depression: a UK-based clinical effectiveness and cost-effectiveness randomised, controlled, superiority trial. *Lancet Psychiatry* 2025; **12:** 433–46.

Supplementary Material

Table of Contents

A. Systematic Review 3

A1. Search String 3

A2. PRISMA Flow Diagram 3

A3. Table of Studies 4

A4. Influence Analysis 5

A5. Graphic Display of Heterogeneity (GOSH) Plot 7

A6. Forest Plots and Pooled Effect Sizes 8

A6.1 MBCT versus TAU 8

A6.2 MBCT versus Active Psychological Control 8

A6.3 MBCT versus TAU (including RESPOND trial) 9

A6.4 MBCT versus active or inactive controls (including RESPOND trial) 9

B. Statistical Analysis Plan (SAP) 10

C. Health Economics Analysis Plan (HEAP) 28

D. Sensitivity Analyses 35

D1. Sensitivity Analysis Taking into Account NHS Talking Therapies Services Effects 35

D2. Sensitivity Analysis Taking into Account Therapist Effects 37

D3. Sensitivity Analysis Taking into Account Different Inclusion Criteria 38

D4. Sensitivity Analysis Taking into Account Size of Assessment Window 40

D6. Sensitivity Analyses with Imputed Missing Data 42

D7. Repeated Measures Analysis 43

D8. Sensitivity Analysis to Handle Post-Randomisation Ineligible Participants 44

D9. Rates of Participants Showing at least 50% Reduction of Depressive Symptomatology (PHQ-9) in MBCT+TAU and TAU at 10-weeks and 34-weeks 45

D10. Summary of Logistic Regression Models Predicting Dichotomous Outcomes at 10-week and 34-week Follow-Up using Baseline Scores as Covariates 46

D11. Linear Regression Models for PHQ-9 Adjusting for Relationship Status (Partner Yes/No) and Randomisation Variables (Depression Severity, Antidepressant Use at Baseline, Site) 47

D12. Line Graph of PHQ-9 Scores at Baseline, 10 Weeks and 34 Weeks 48

E. Health Economic Analyses 49

E1. Unit Costs and Sources Used for the Economic Evaluation 49

E2. Use of Health and Social Care Services 50

E3. Medication Use 51

E4. Health and Social Care Perspective (Primary Analysis) 52

E4.1 Health and Social Care Perspective (Complete Case) 52

E4.2 Health and Social Care Perspective (Multiple Imputation) 58

E5. Health and Social Care and Productivity Costs Perspective 59

E5.1 Health and Social Care and Productivity Costs Perspective (Complete Case) 59
E5.2 Health and Social Care and Productivity Costs Perspective (Multiple Imputation) 61

F. Modifications to Original MBCT Treatment Manual..63

G. Serious Adverse Events 69

H. Internal Consistency of Outcome Measures 70

I. Patient Identification Centres (PIC) 71

J. PPI Involvement...72

A. Systematic Review

A1. Search String

PubMed
("depression"[MeSH Terms] OR "depressive disorder, major"[MeSH Terms] OR "MDE"[Title/Abstract] OR "MDD"[Title/Abstract] OR "dysthymia"[Title/Abstract] OR "dysthymic"[Title/Abstract]) **AND** ("chronic"[Title/Abstract] OR "persistent"[Title/Abstract] OR "treatment-resistant"[Title/Abstract] OR "non-responders"[Title/Abstract] OR "non-response"[Title/Abstract] OR "non-remitted"[Title/Abstract] OR "dysthymia"[Title/Abstract] OR "dysthymic"[Title/Abstract]) **AND** ("MBCT"[Title/Abstract] OR "mindfulness-based cognitive therapy"[Title/Abstract]) **AND** ("randomized controlled trial"[All Fields] OR "randomised controlled trial"[Title/Abstract] OR "randomized-controlled trial"[Title/Abstract] OR "randomised-controlled trial"[Title/Abstract] OR "RCT"[Title/Abstract] OR "randomized"[Title/Abstract] or "randomised"[Title/Abstract] OR "randomly allocated"[Title/Abstract] OR "randomly assigned"[Title/Abstract])

A2. PRISMA Flow Diagram

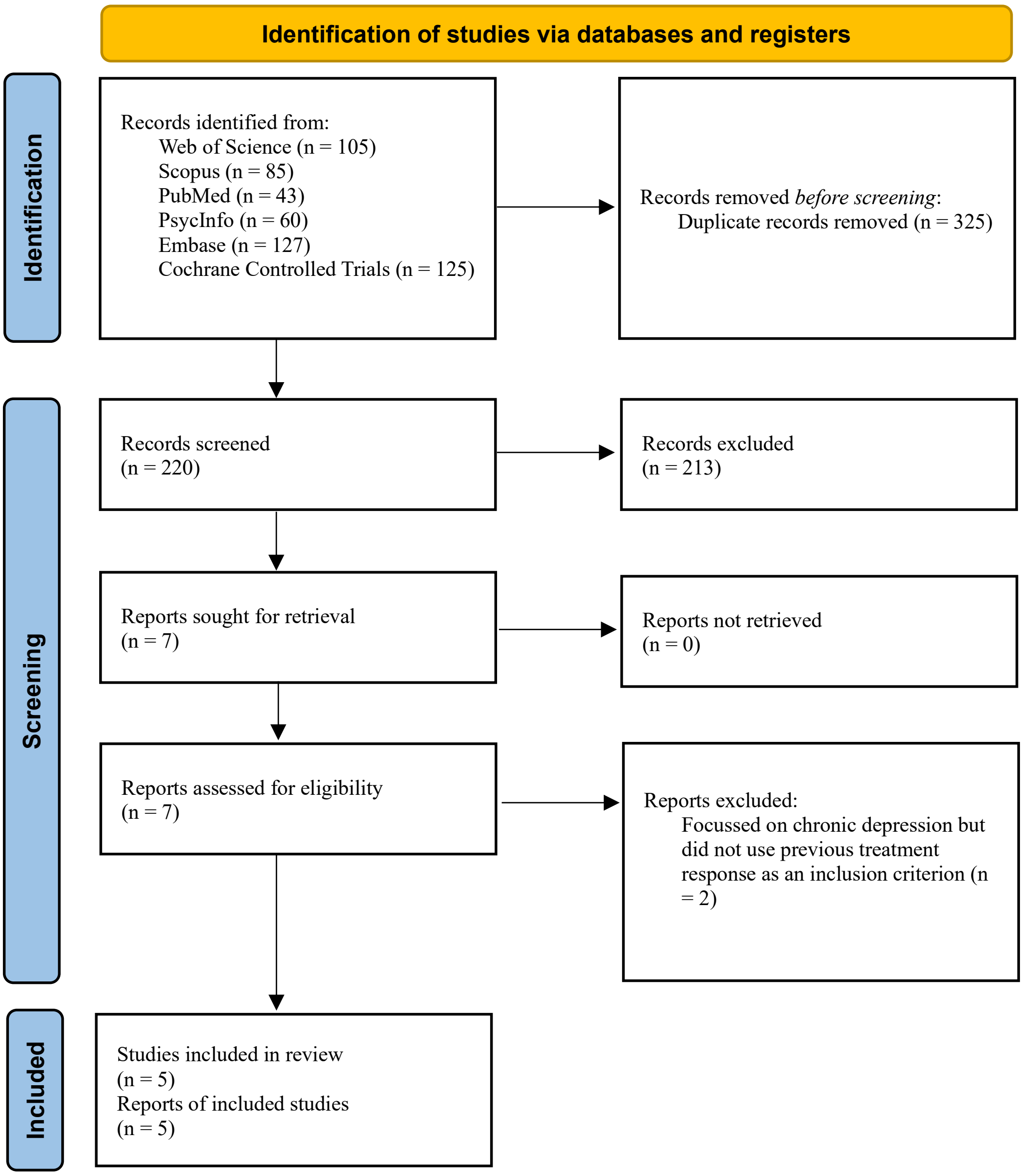

A3. Table of Studies

Study	Conditions (N analysed)	Assessments	Primary Outcome	Sample
Chiesa et al. (2015)	MBCT (n = 23) vs<br>Psych-Education (n = 20)	T0: Baseline<br>T1: 4 weeks post-randomisation<br>T2: 8 weeks post-randomisation (post-treatment)<br>T3: 17 weeks post-randomisation (follow-up)<br>T4: 26 weeks post-randomisation (follow-up)	Hamilton Rating Scale for Depression	Non-responders (pharmacological therapy)
Cladder-Micus et al. (2018)	MBCT (n = 44) vs<br>TAU (n = 52)	T0: Baseline<br>T1: 8 weeks post-randomisation (post-treatment)	Inventory of Depressive Symptomatology (Self-Report)	Treatment-resistant depression (pharmacological and psychological treatment)
Eisendrath et al. (2016)	MBCT (n = 87) vs<br>Health Enhancement Program (n = 86)	T0: Baseline<br>T1: 8 weeks post-randomisation (post-treatment)<br>T2: 24 weeks post-randomisation (follow-up)<br>T3: 36 weeks post-randomisation (follow-up)<br>T4: 52 weeks post-randomisation (follow-up)	Hamilton Rating Scale for Depression	Treatment-resistant depression (pharmacological treatment)
Foroughi et al. (2020)	MBCT (n = 10) vs<br>TAU (n = 9)	T0: Baseline<br>T1: 8 weeks post-randomisation (post-treatment)<br>T2: 12 weeks post-randomisation (follow-up)	Hamilton Rating Scale for Depression	Treatment-resistant depression (pharmacological treatment)
Garcia et al. (2023)	MBCT (n = 29) vs<br>LMP (n = 34) vs<br>Placebo/TAU (n = 31)	T0: Baseline<br>T1: 8 weeks post-randomisation (post-treatment)<br>T2: 26 weeks post-randomisation (follow-up)<br>T3: 52 weeks post-randomisation (follow-up)	Beck Depression Inventory - II	Treatment-resistant depression (pharmacological treatment)

A4. Influence Analysis

A Baujat plot and influence analyses (including computation of externally standardized residuals, DFFITS values, Cook's distances, covariance ratios, estimated heterogeneity as measured by τ^2 and Cochran's Q, if study k is removed) indicated that the study by Foroughi et al. (2020) contributed heavily to overall heterogeneity. As this study clearly represented an outlier (g = -8·22, SE = 1·47), pooled effect sizes were computed with the study removed.

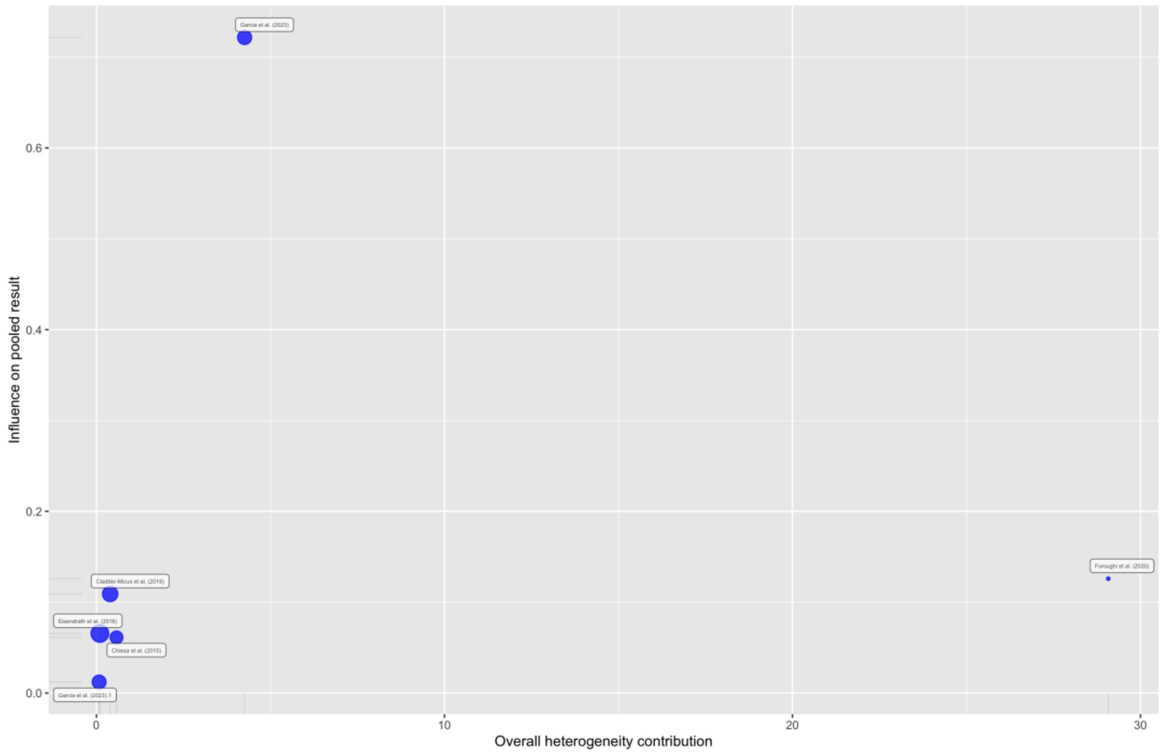

Supplementary Figure A4.1

Baujat plot

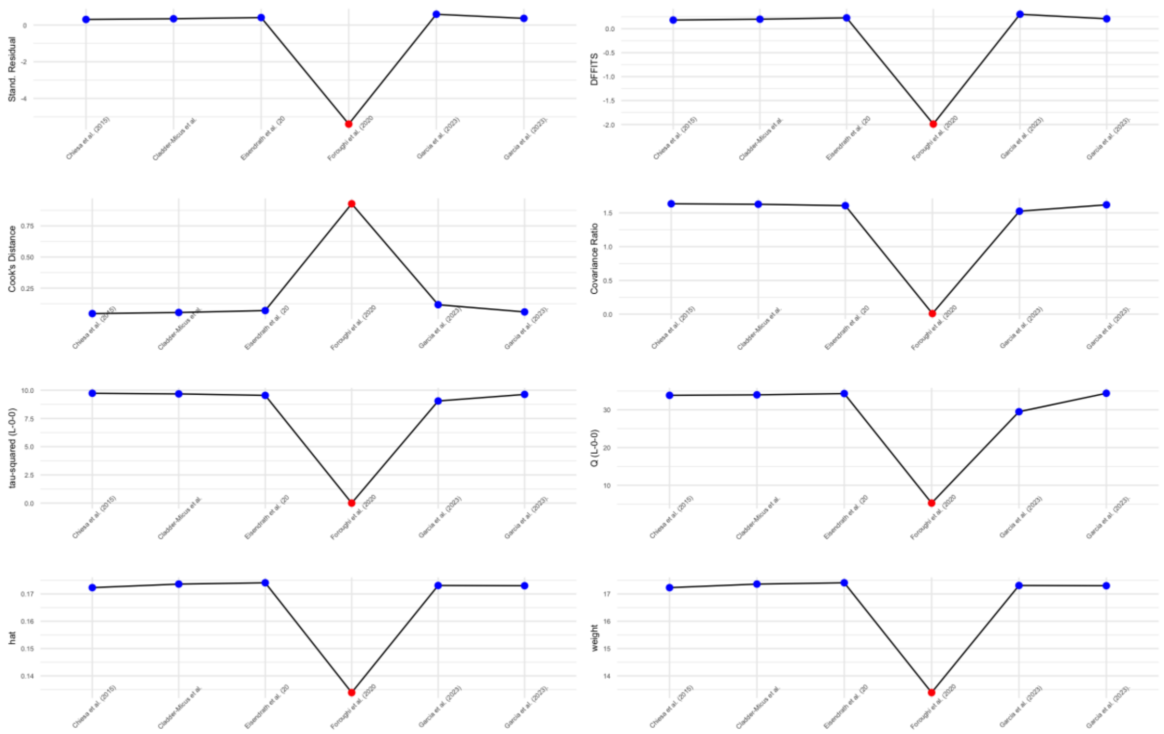

Supplementary Figure A4.2

Influence statistics

A5. Graphic Display of Heterogeneity (GOSH) Plot

A GOSH plot depicting pooled effect sizes and heterogeneity scores (I2) of all possible combinations of studies showed two distinct clusters suggesting different effect size populations in the data. We therefore decided to compute pooled effect sizes separately for studies comparing MBCT against treatment as usual or placebo and studies comparing MBCT against active psychological control interventions.

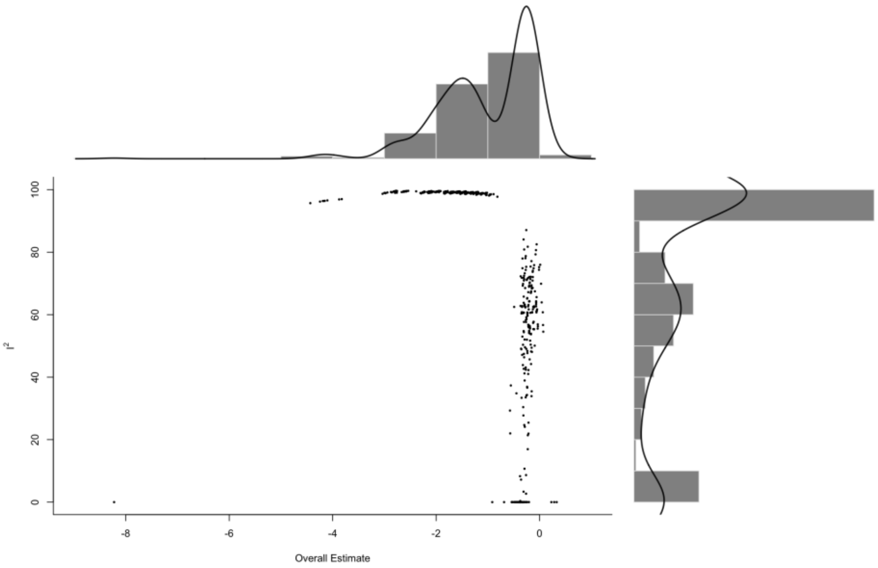

Supplementary Figure A5.1

GOSH plot

A6. Forest Plots and Pooled Effect Sizes

A6.1 MBCT versus TAU

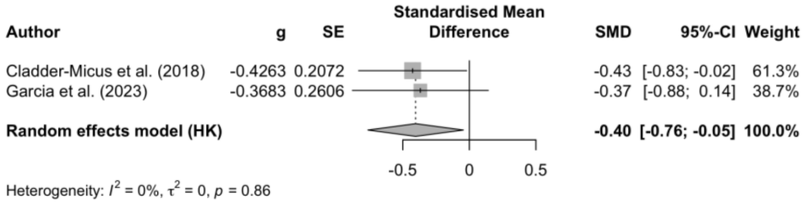

Supplementary Figure A6.1

Forest plot of studies comparing MBCT and TAU

A6.2 MBCT versus Active Psychological Control

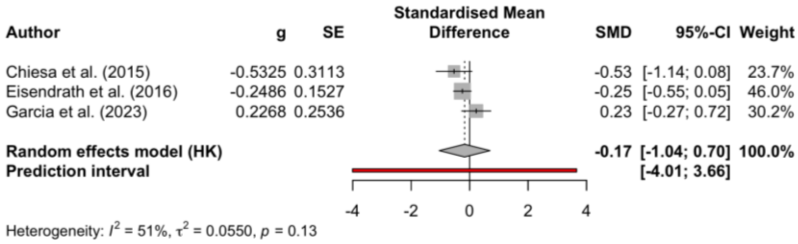

Supplementary Figure A6.2

Forest plot of studies comparing MBCT and active psychological controls

A6.3 MBCT versus TAU (including RESPOND trial)

Adding the results of the RESPOND trial to the meta-analysis of studies comparing MBCT versus TAU consolidates the findings indicating a small to moderate effect size.

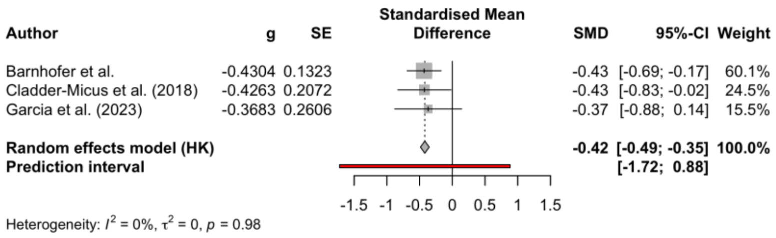

Supplementary Figure A6.3

Forest plot of studies comparing MBCT and TAU (including RESPOND trial)

A6.4 MBCT versus active or inactive controls (including RESPOND trial)

Entering studies of MBCT for treatment non-responders across studies with inactive and active controls and including the results from the RESPOND trial in a random effects meta-analysis yields a small effect. However, heterogeneity is high (as discussed further above).

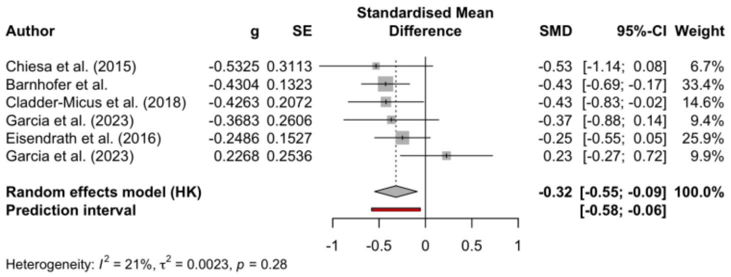

Supplementary Figure A6.4

Forest plot of studies comparing MBCT and active or inactive controls (including RESPOND trial)

B. Statistical Analysis Plan (SAP)

A randomised controlled trial to investigate the clinical effectiveness and cost-effectiveness of Mindfulness-Based Cognitive Therapy (MBCT) for depressed non-responders to Increasing Access to Psychological Therapies (IAPT) high-intensity therapies

[Acronym: MBCT for IAPT Non-Responders (RESPOND)]

Statistical Analysis Plan (SAP)

This Statistical Analysis Plan (SAP) was finalised on 21 April 2023; version number 1.0.

This SAP builds on the information provided in the Study Protocol v3.0 28/10/2022. The trial is registered at ISRCTN under number 17755571 02/03/2021.

Revisions to this SAP and details of the revisions are provided below.

SAP revision number	Date of revision *(DD-MMM-YYYY)*	Timing of SAP revision in relation to analyses, eg: before interim analysis	Details of the revision	Justification for the revision
1		No revisions to date		

This SAP was produced by members of the trial team as described in the table:

Name	Affiliation(s)	Role
Dr Fiona Warren	University of Exeter Clinical Trials Unit	Senior statistician

This version of the SAP has been approved by the Trial Management Group and is signed below to confirm this.

	Author of SAP	Senior statistician	Chief Investigator
**Name**	Dr Fiona Warren	Dr Fiona Warren	Prof Thorsten Barnhofer
**Signature**	[illegible]	[illegible]	[illegible]
**Date (DD-MM-YYYY)**	21/04/2023	21/04/2023	21/04/2023

1

Table of Contents

1. Introduction 4
1.1 Background and rationale 4
1.2 Objectives 4
Aims 4
Objectives 4
Hypotheses 5
Estimands 5
1.3 Study methods 5
Trial design 5
Randomisation 6
Sample Size 6
Framework 6
Statistical interim analyses and stopping guidance 6
Timing of final analysis 6
Timing of outcome assessments 6
1.4 Statistical Principles 6
Confidence intervals and p-values 6
Intervention adherence and protocol deviations 7
Analysis populations 7
1.5 Trial population 7
Screening data 7
Eligibility 7
Recruitment 8
Withdrawal/follow-up 10
Baseline patient characteristics 10
1.6 Statistical Analysis 10
Outcome definitions 10
Analysis methods 13
Additional analyses and sensitivity analyses 13
Harms 15
Statistical software 15
2. Related documents 15
3. References 16
Appendix 18
A1 Adherence to MBCT practice 18

A2 Example tables.. 18

1. Introduction

1.1 Background and rationale

Major Depression represents a pressing challenge for health care. The disorder is not only highly prevalent – 10.9 percent of the adult population in England suffered from an episode of depression in 2014 [1] – but also shows many characteristics of a progressive disease. If left untreated it tends to become more recurrent and chronic over time [2], with even residual levels of symptoms conferring a significantly increased risk for future relapse [3]. There is evidence for functional decline as the disorder accelerates [4], and physiological changes underlying its progression have been linked with a significantly increased risk for a broad range of physical and neurodegenerative disorders [5]. It is important therefore to treat depressive episodes sufficiently. Increasing Access to Psychological Therapies (IAPT) services have been introduced as a means of providing patients with evidence-based psychological therapies in a timely manner. The services were intended to reach an access rate of 25% of the population in 2020/21. However, outcome data indicate that about 50% of the patients who receive high-intensity therapy do not recover fully.

Recent research has brought promising evidence that Mindfulness-Based Cognitive Therapy (MBCT) [6] can have significant beneficial effects in patients with acute and more persistent forms of the disorder [7, 8], and particularly in those who have not responded to previous interventions [9]. The aim of this project is to test whether MBCT could serve as an effective and cost-effective intervention for patients who have not responded to IAPT high-intensity therapy.

If successful, the proposed research would provide the evidence necessary for adoption of MBCT for non-responders within IAPT and would thus help to justify the use of an easy to implement and much needed treatment option for a considerable proportion of patients who are currently not receiving sufficient support. We compare MBCT as delivered via videoconferencing to treatment-as-usual (TAU) in IAPT high-intensity treatment non-responders in a definitive clinical trial. TAU was chosen as comparator as it is reflective of the current state of care. We will test the immediate effects of the intervention on depressive symptomatology as well as whether effects on symptomatology can be sustained over a period of six months. Further information on the background to the study can be found in the Study Protocol v3.0 28/10/2022.

1.2 Objectives

Aims

To establish the

(a) clinical effectiveness in terms of reductions in depressive symptomatology and

(b) cost-effectiveness of MBCT as a psychotherapeutic treatment option compared with TAU for depressed patients who have not responded sufficiently to high intensity evidence-based treatments within the IAPT care pathway.

Objectives

(a) To undertake a definitive randomised controlled trial (RCT) of the MBCT intervention versus TAU to confirm clinical effectiveness of the treatment in depressed non-responders to high-intensity evidence-based treatments within the IAPT care pathway, and

(b) To use the data from the RCT to conduct a cost-utility and cost-effectiveness analysis to provide information on whether or not the MBCT intervention is worthwhile economically

Hypotheses

We hypothesise that:

(a) participants who receive MBCT will show significantly stronger reductions in depressive symptomatology measured using Patient Health Questionnaire-9 (PHQ-9) [10] than participants who receive TAU both at 10 weeks post-randomisation (post-treatment; secondary outcome) and at 34 weeks post-randomisation (primary outcome); and

(b) the MBCT intervention will be cost-effective, either in terms of reductions in costs elsewhere in the health system or in improvements in outcomes.

Estimands

Using the estimands framework [11,12], our target estimands are set out below.

Table 1 Estimands for RESPOND trial

Population	Patients aged 18 or older who have not responded to high-intensity IAPT interventions for depression (PHQ-9 score≥10 after 12 sessions), but do not meet eligibility criteria for secondary care services
Treatment conditions	Intervention: MBCT<br>Control: TAU
Outcome variable	PHQ-9 at 34-week follow-up
Handling of intercurrent events	1. Treatment policy<br>2. Principal stratum (CACE analyses – see Section 1.4)
Population level summary measure	Between group mean difference

1.3 Study methods

Trial design

The study population comprises patients aged 18 or older who have not responded to high-intensity IAPT interventions for depression, but do not meet eligibility criteria for secondary care services.

Interventions

A two-arm trial, across 3 sites, will randomise 234 participants in a 1:1 ratio to receive either MBCT or to continue with TAU, with TAU providing a comparator that is reflective of the current state of care (and in most cases will entail continued use of antidepressant medication).

MBCT intervention

The intervention will be delivered by trained MBCT therapists together with an assistant to groups of about 13 patients (minimum 8 and maximum of 16) using videoconferencing on a secure online platform. This is a change from the original intention of delivery face-to-face due to Covid-19; this amendment was documented in Protocol V01 (14.10.2020). Participants will attend sessions through internet connection from their home or another place of their choosing. MBCT consists of eight weekly group-based sessions and participants are asked to engage in home practice for about an hour per day using guided meditation audio recordings, with attendance and practice monitored following previously established practices. Manual adherence and treatment fidelity will be rated based on the recordings of the online intervention sessions using methods established in our previous trials using the MBCT Adherence Scale [13] and MBI-TAC [14].

TAU comparator arm

Participants in the TAU condition will be asked to continue with their usual care and follow the regimens suggested by their GP or mental health professional, which in most cases will consist of continuing use of antidepressant medication. Following previous practice in our trials [15], TAU participants will be invited to an interview to prevent tendencies towards 'resentful demoralisation' and highlight the importance of their contribution. The pre-class interview for the MBCT courses will also be conducted via videoconferencing.

Randomisation

Participants will be allocated to either MBCT or TAU, at a ratio of 1:1, through remote randomisation at the UKCRC-registered Exeter Clinical Trials Unit (ExeCTU), following informed consent, completion of baseline assessment and enrolment in the trial. Randomisation will use minimisation on depression severity (PHQ-9<19 versus ≥19), antidepressant use at baseline and recruitment site.

Sample Size

Details of the sample size calculation are provided in the study protocol. The sample size calculation is based on the primary outcome: PHQ-9 measured at 34 weeks post-randomisation. To detect an MCID of 2.59, using a standard deviation of 5.4, with 90% power at an alpha level of .05, 186 participants are required. Considering a rate of attrition of 20%, conservatively estimated to be above that observed in our previous research [15], we will aim to recruit a total sample of 234 participants (117 in each arm, 78 per site).

Framework

This trial is a fully powered definitive trial that seeks to evaluate superiority of the MBCT intervention over TAU.

Statistical interim analyses and stopping guidance

No interim analyses will be performed for efficacy or harms. As the intervention is considered to be low risk to participants, there are no formal guidelines for early termination of the trial due to potential for harm to participants. All adverse events and serious adverse events will be reported to the TSC and DMEC for their consideration; if the TSC and DMEC consider that there is sufficient cumulative evidence of harm to participants due to the intervention(s), the trial will be discontinued. Also, there are no guidelines for early termination due to futility (inability to achieve statistical significance for a treatment effect) or achieving significant results prior to full data analysis.

Timing of final analysis

We anticipate performing all analysis following final database lock, when all follow-up data (up to and including 34-week follow-up) has been entered and cleaned. Timing of each observation will be counted from the date of baseline measurement for the individual participant.

Timing of outcome assessments

We will measure outcomes at baseline, 10-week and 34-week follow-up post-randomisation. A 7-day window will be available for patients to complete the follow-up assessments and participants who do not respond within this time window will be prompted weekly to respond but no longer than until the end of a 4-week period.

1.4 Statistical Principles

Confidence intervals and p-values

All inferential analyses will be reported using 95% confidence intervals and p-values, with the threshold for statistical significance set at 0.05. No formal testing for multiple comparisons will be

performed (i.e. for multiple comparisons across the primary and secondary outcomes); the p-values for the primary analysis of the primary outcome (ITT analysis of PHQ-9 at 34-week follow-up) will be interpreted first, and the p-values for the secondary outcomes will be interpreted in the light of the overall results.

Intervention adherence and protocol deviations

Intervention adherence

To inform the Complier Average Causal Effect (CACE) analysis, a participant in the MBCT group will be considered a `complier' if a minimum of four sessions are attended; all participants in the control group will be considered as `compliers'.

Discontinuation from the study

Participants are free to withdraw their participation at any point. If a participant in either arm indicates that they wish to discontinue the trial they will not be contacted further by the research team, other than to invite them to take part in a brief written survey to ascertain their reasons for not taking part.

In the MBCT arm of the trial, a participant may discontinue therapy but remain in the trial. In order to enable intention to treat analyses, we will still ask participants who opt to discontinue therapy at any point to take part in assessments, should they be willing to contribute to the research in this way.

Analysis populations

Although the TAU condition is unlimited, it is practically highly unlikely for TAU participants to receive MBCT if this was not the participant's randomised allocation. The only possibility for a participant failing to receive the randomised intervention is if the participant did not adhere in the MBCT group. We do not expect an `as treated' analysis to be required as it is highly unlikely for a participant in the control group to receive the MBCT intervention.

1.5 Trial population

Screening data

Potential participants who believe that they may be eligible for the trial will be requested to consent to a further screening procedure and to participate in the trial if they are eligible. Data will be retained for participants who are screened but found to be ineligible. We will report data on age and sex for people who are screened, and found to be eligible, but who do not participate further in the trial.

Eligibility

The study population comprises patients aged 18 and older who have not responded to high-intensity IAPT interventions for depression, but do not meet eligibility criteria for secondary care services.

Inclusion criteria will be:

1) non-response to a minimal effective dose of high intensity treatment for depression (primary presenting problem) in IAPT (at least 12 sessions, in line with NICE guideline suggestions) defined in line with the caseness threshold adopted by IAPT as a PHQ-9 score of 10 or higher [10]

2) meeting criteria for a current episode of Major Depression according to DSM-5 as assessed through the Mini International Neuropsychiatric Interview for DSM-5 (MINI 7.0.2) [16] along with a current PHQ-9 score of 10 or higher

3) age 18 or older, and

4) access to a working internet connection and equipment to participate in videoconferencing assessments and interventions.

Exclusion criteria will be:

1) based on the judgment of their IAPT therapist they are eligible for, would be seen by, and their needs would be best met by secondary care specialist services

2) they present with a level of risk to self or others that cannot be safely managed in a primary care service context (i.e. active suicidal plans), a history of psychosis or psychotic symptoms, a current episode of mania, alcohol or substance abuse or dependence within the past 3 months, a current post-traumatic stress disorder, an obsessive-compulsive disorder or an eating disorder

3) they suffer from any other significant disease or disorder that may either put the participant at risk because of participation in the trial, or may influence the result of the trial, or the participant's ability to participate in the trial

4) if they have an insufficient ability to understand or read English.

Patients who are currently taking antidepressant medication will be allowed into the trial and medication use will be documented for statistical analysis. Medication use will be included as a stratification variable in the minimisation algorithm for randomisation.

According to the IAPT database, the majority of patients who receive high intensity psychological treatment will also have received treatment with antidepressant medication, and most of these patients will therefore meet consensus criteria for treatment resistance. We will compare the sociodemographic characteristics of our sample against the characteristics of the wider group of people attending the collaborating IAPT services in order to judge representativeness of the sample (Table 3).

Recruitment

Information on participant recruitment to the trial is described fully in the trial protocol.

IAPT patients who are potentially interested in taking part will be contacted by the researchers via telephone for an initial screening to assess eligibility and to provide further information on the research. Baseline assessments will then be completed. Eligible, fully informed and consenting participants will then be entered into the study and randomised (see Figure 1).

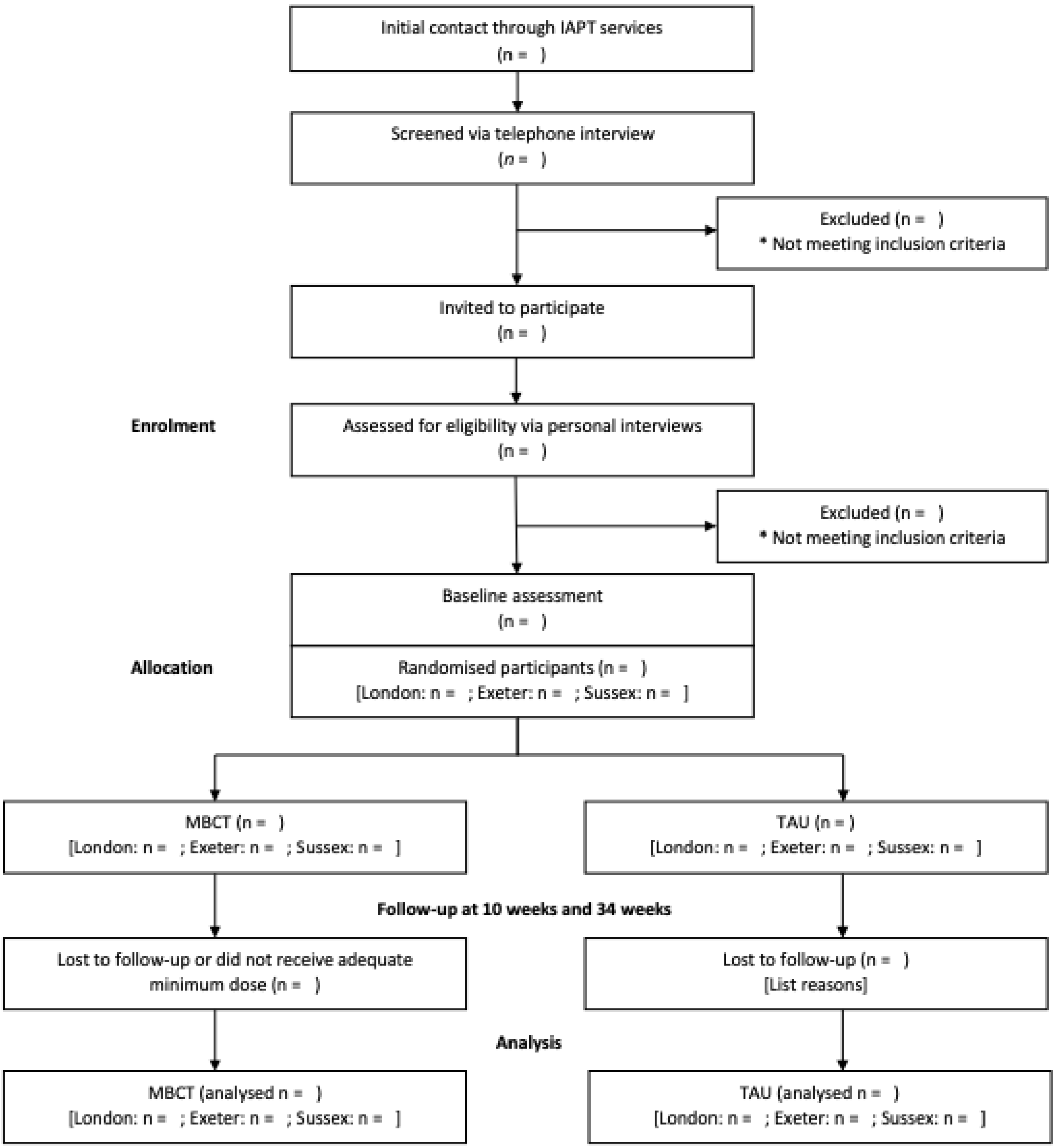

Figure. 1 CONSORT diagram describing flow of participants through the study

Withdrawal/follow-up

Participant withdrawal (from treatment in the MBCT group and follow-up for all participants) will be reported using a CONSORT flow diagram. We will report descriptively the baseline characteristics of participants who are lost to follow-up at 34 weeks, and will also explore baseline characteristics that are predictors of missing PHQ-9 data at 34 weeks, to inform the imputation modelling.

Baseline patient characteristics

Participant characteristics assessed as part of the Mini International Neuropsychiatric Interview (MINI) at baseline will allow us to make comparisons between eligible patients who declined to participate (if any), and those patients who participated in the trial (Table A1).

1.6 Statistical Analysis

Patient characteristics will be compared across the MBCT and TAU arms (Table A2).

Outcome definitions

Primary outcome

The primary clinical outcome will be reductions in depression symptomatology as assessed using the PHQ-9 [10]. The PHQ-9 is a widely used self-report measure of depression that represents an integral part of the management of depression in the IAPT pathway and has good psychometric properties. The primary timepoint for outcome measures will be 34 weeks post-randomisation. Hence, the primary outcome will be PHQ-9 scores at 34-week follow-up.

Secondary outcomes

Secondary outcomes include PHQ-9 measured at 10 weeks post-randomisation, and other clinical outcomes measured at 10-week and 34-week follow-up. Other clinical secondary outcome measures will include the Generalized Anxiety Disorder Questionnaire (GAD-7) [17], the Phobia Scale, and the Work and Social Adjustment Scale, all from the IAPT minimum data set (IAPT Toolkit, 2008/9), along with the Warwick-Edinburgh Mental Wellbeing Scale (WEMWBS) [18], Experiences Questionnaire (EQ) Decentering Scale [19] and Five Factor Mindfulness Questionnaire (FFMQ) [20]. We will also track symptoms weekly during the MBCT intervention using the PHQ-9.

A series of binary outcomes will also be derived, based on PHQ-9 and/or GAD-7 at 34-week follow-up (Table 1). Definitions of these outcomes are based on conventions used by IAPT [21].

Table 2 Binary outcome measures derived from PHQ-9 and GAD-7 at 34-week follow-up

Measure #	Measure name	Underlying continuous outcome(s)	Measure definition	Notes
**Outcomes based on PHQ-9 only**				
1	Recovery	PHQ-9	Change from a score ≥10 at baseline to ≤9 at follow-up	Only applies to participants with a PHQ-9 score ≥10 at baseline
2	Reliable recovery	PHQ-9	Change from a score ≥10 at baseline to ≤9 at follow-up plus reduction in score by ≥6 units	Only applies to participants with a PHQ-9 score ≥10 at baseline
3	Reliable improvement	PHQ-9	Reduction in score by ≥6 units	Applies to all participants
4	Deterioration	PHQ-9	Increase in score	Applies to all participants
5	Reliable deterioration	PHQ-9	Increase in score by ≥6 units	Applies to all participants
**Outcomes based on GAD-7 only**				
6	Deterioration	GAD-7	Increase in score	Applies to all participants
7	Reliable deterioration	GAD-7	Increase in score by ≥4 units	Applies to all participants
**Outcomes based on PHQ-9 and GAD-7**				
8	Recovery	PHQ-9; GAD-7	Participant scored above the clinical threshold on depression and/or anxiety, i.e. PHQ-9 ≥10 at baseline AND/OR GAD-7 ≥8 at baseline<br>Recovery occurs if the participant scores below the clinical threshold at follow-up on depression AND anxiety, i.e. PHQ-9 score ≤9 AND GAD-7 ≤7 at follow-up	Applies to all participants; however, not all participants will have GAD-7 ≥8 at baseline
9	Reliable recovery	PHQ-9; GAD-7	Participant shows reliable improvement as defined below and scores below the clinical threshold at follow-up on depression AND anxiety, i.e. PHQ-9 score ≤9 AND GAD-7 ≤7 at follow-up.<br>Reliable recovery combines reliable improvement and recovery	Applies to all participants; however, not all participants will have GAD-7 ≥8 at baseline
10	Reliable improvement	PHQ-9; GAD-7	Criterion 1: Reliable improvement in PHQ-9 AND reliable improvement in GAD-7; OR	Applies to all participants

			Criterion 2: Reliable improvement in PHQ-9 AND no reliable change in GAD-7; OR<br>Criterion 3: No reliable change in PHQ-9 AND reliable improvement in GAD-7	
11	No reliable change[1,2]	PHQ-9; GAD-7	Criterion 1: No reliable change in PHQ-9 AND no reliable change in GAD-7 OR<br>Criterion 2: Reliable improvement in PHQ-9 AND reliable deterioration in GAD-7 OR<br>Criterion 3: Reliable deterioration in PHQ-9 AND reliable improvement in GAD-7	Applies to all participants
12	Reliable deterioration	PHQ-9 GAD-7	Criterion 1: Reliable deterioration in PHQ-9 AND reliable deterioration in GAD-7 OR<br>Criterion 2: Reliable deterioration in PHQ-9 AND no reliable change in GAD-7 OR<br>Criterion 3: No reliable change in PHQ-9 AND reliable deterioration in GAD-7	Applies to all participants

[1]No reliable change in PHQ-9: change in score <6 units. [2]No reliable change in GAD-7: change in score <4 units.

Analysis methods

Primary analysis

The primary analysis approach will use the intention-to-treat principle (all participants will be included in the analysis according to their randomised allocation irrespective of the treatment actually received) including observed data only. All outcomes will be reported descriptively at baseline, and at 10 and 34 weeks' follow-up. Descriptive data will be reported for the overall sample and for each site individually. Continuous outcomes will be analysed using linear regression models. The binary outcomes will be analysed using logistic regression. All analyses will adjust for participant covariates (depression severity (PHQ-9<19 versus ≥19), antidepressant use at baseline and recruitment site) used in randomisation, with adjustment for baseline scores for continuous outcomes. We will assess other participant characteristics at baseline (including number of previous episodes), and will consider performing a sensitivity analysis with adjustment for any covariates that are found to be substantively unbalanced for the ITT analysis using observed data only, should such covariates be considered predictive of outcome. Inferential between group comparisons (MBCT vs TAU) for the primary and all secondary outcomes will be performed at 34-week follow-up.

Primary analyses will include all data collected within the overall window for each follow-up time, and will be performed by a statistician who is blinded to intervention allocation. Following presentation of the results of the primary analyses and unblinding of the trial team, the remaining additional and sensitivity analyses will be performed unblinded.

Additional analyses and sensitivity analyses

With the exception of the complier average causal effect (CACE) analysis described below, all sensitivity analyses will use the ITT approach. With the exception of the multiple imputations analysis described below, all sensitivity analyses will use observed data only.

Assessment of therapist effects

To address the potential effects of clustering by therapist, we will perform mixed effects linear regression models for the primary and secondary (continuous and binary) outcomes with a random effect on therapist. In addition, we will perform linear or logistic regression models adding therapist seniority as a predictor for the primary outcome and all secondary outcomes for the MBCT group only.

Assessment of IAPT effects

To address the potential effects of clustering by IAPT service, we will perform mixed effect regression models for the primary and secondary (continuous and binary) outcomes with a random effect on IAPT service.

Exploration of different inclusion criteria

To explore effects under conditions of different inclusion criteria, we will perform a sensitivity analysis excluding participants showed reliable improvement in PHQ-9 (i.e. a reduction by 6 points on more on PHQ-9) during IAPT treatment. This sensitivity analysis will be performed using regression modelling for the primary and secondary outcomes.

Inclusion of data collected outside the 7-day time window

We will perform a sensitivity analysis for the primary outcome only to include data collected during the 7-day data collection window only.

Complier average causal effect analysis

As a sensitivity analysis, we will perform a CACE analysis, to estimate the treatment effect while accounting for non-adherence to treatment. A participant in the intervention arm will be considered to be 'complier', if a minimum of four treatment sessions were attended. A 2-stage least squared instrumental variable regression model will be used, for the purpose of identifying those participants in the TAU group who would be `compliers' had they been allocated to MBCT, and comparing the compliers in both groups. A CACE analysis will be performed for the PHQ-9 and all continuous secondary outcomes.

Imputing missing data

A sensitivity analysis will use multiple imputation to impute missing outcome data for the primary outcome and all secondary continuous outcomes at 34 weeks. Multiple imputation using chained equations (MICE) will be used; the imputation algorithm will include baseline characteristics that are found to be predictive of missingness of the primary outcome and outcome data reported at 10-week follow-up, as well as treatment arm and minimisation variables. Logistic regression will be used to determine characteristics associated with missing primary outcome data (PHQ-9 at 34-week follow-up). Predictive mean matching will be the method for imputing individual scores; the number of imputed datasets will be determined by the percentage of participants that have missing primary outcome data. Observed and imputed data will be used to perform a sensitivity analysis of the inferential between group comparisons at 34 weeks.

Repeated measures analysis

A repeated measures analysis will be performed for the primary outcome and continuous secondary outcomes, using a mixed effects linear regression model with a random effect on participant (i.e. observations at different timepoints will be nested within participant), and including an interaction term between treatment group and timepoint. All other predictors will be included as fixed effects within the model. This model will include participants with follow-up data reported for at least one follow-up time, using observed data according to the ITT principle.

Sensitivity analysis to handle post-randomisation ineligible participants

It was noted on 22 November 2022 that an error had been made in the algorithm for detection of potentially eligible participants from one of the Sussex Partnership Foundation Trust IAPT services associated with the Sussex site. The algorithm was intended to detect all IAPT patients who had received therapy for depression and had a PHQ-9 score of 10 or more at their *final* IAPT session. However, the algorithm erroneously included all those who had scored a PHQ-9 score of 10 or higher *at any session during their therapy*, which rendered 14 participants ineligible. On further investigation, a total of 24 participants were found to be ineligible post-randomisation due to this error in coding. In addition, a participant was found to be ineligible due to not having received the recommended number of 12 therapy sessions during their initial IAPT treatment, and one participant who was already ineligible due to having a PHQ-9 at final session of less than 10, and also received fewer than 12 therapy sessions. Overall, 25 participants were randomised into the trial despite not having met required inclusion criteria. However, it was considered that these participants met all other eligibility criteria. Hence it is anticipated that these participants would be very similar to those that were eligible based on the inclusion criteria.

To address this issue, the 25 post-randomisation ineligible participants will be excluded from the primary analyses, but will be included in a sensitivity analysis for the primary outcome only.

Harms

Adverse Events (AEs) will be reported at fixed timepoints, set by frequency of DSMC meetings (to be determined). For definitions of AEs, see the protocol.

Statistical software

All analyses will be carried out using Stata v17.0 or later.

2. Related documents

#	Document Number	Document Title	Source
1	Data Management Plan		
2	Trial Master File		
3	Statistical Master File		
4	SOP_019	Deviations, Misconduct and Serious Breaches of GCP and(or) the Protocol	

3. References

1. NHS Digital. Mental Health and Wellbeing in England: Adult Psychiatric Morbidity Survey 2014. 2016.

2. Solomon DA, Keller MB, Leon AC, Mueller TI, Lavori PW, Shea MT, et al. Multiple recurrences of major depressive disorder. Am J Psychiatry. 2000;157(2):229-33.

3. Judd LL, Akiskal HS, Maser JD, Zeller PJ, Endicott J, Coryell W, et al. Major depressive disorder: a prospective study of residual subthreshold depressive symptoms as predictor of rapid relapse. J Affect Disord. 1998;50(2-3):97-108.

4. Moylan S, Maes M, Wray NR, Berk M. The neuroprogressive nature of major depressive disorder: pathways to disease evolution and resistance, and therapeutic implications. Mol Psychiatry. 2013;18(5):595-606.

5. Bhattacharya R, Shen C, Sambamoorthi U. Excess risk of chronic physical conditions associated with depression and anxiety. BMC Psychiatry. 2014;14(1):10.

6. Segal ZV, Williams JMG, J T. Mindfulness-Based Cognitive Therapy for Depression. 2nd ed: New York: Guilford; 2013.

7. Barnhofer T, Crane C, Hargus E, Amarasinghe M, Winder R, Williams JM. Mindfulness-based cognitive therapy as a treatment for chronic depression: A preliminary study. Behav Res Ther. 2009;47(5):366-73.

8. Winnebeck E, Fissler M, Gärtner M, Chadwick P, Barnhofer T. Brief training in mindfulness meditation reduces symptoms in patients with a chronic or recurrent lifetime history of depression: A randomized controlled study. Behav Res Ther. 2017;99:124-30.

9. Eisendrath SJ, Gillung E, Delucchi KL, Segal ZV, Nelson JC, McInnes LA, et al. A Randomized Controlled Trial of Mindfulness-Based Cognitive Therapy for Treatment-Resistant Depression. Psychother Psychosom. 2016;85(2):99-110.

10. Kroenke K, Spitzer R, Williams J. The PHQ-9: validity of a brief depression severity measure. J Gen Intern Med. 2001;16(9):606-13.

11. Clark TP, Kahan BC, Phillips A, White I, Carpenter JR. Estimands: Bringing clarity and focus to research questions in clinical trials. BMJ Open. 2022;12(1):e052953.

12. Cro S, Kahan BC, Rehal S, Chis Ster A, Carpenter JR, White IR, Cornelius VR. Evaluating how clear the questions being investigated in randomised trials are: Systematic review of estimands. BMJ. 2022;378:e070146.

13. Segal ZV, Teasdale JD, Williams JM, Gemar MC. The mindfulness-based cognitive therapy adherence scale: inter-rater reliability, adherence to protocol and treatment distinctiveness. Clinical Psychology & Psychotherapy. 2002;9(2):131-8.

14. Crane RS, Eames C, Kuyken W, Hastings RP, Williams JM, Bartley T, et al. Development and validation of the mindfulness-based interventions - teaching assessment criteria (MBI:TAC). Assessment. 2013;20(6):681-8.

15. Williams JM, Crane C, Barnhofer T, Brennan K, Duggan DS, Fennell MJ, et al. Mindfulness-based cognitive therapy for preventing relapse in recurrent depression: a randomized dismantling trial. J Consult Clin Psychol. 2014;82(2):275-86.

16. Sheehan DV, Lecrubier Y, Sheehan KH, Amorim P, Janavs J, Weiller E, et al. The Mini-International Neuropsychiatric Interview (M.I.N.I.): The development and validation of a structured diagnostic psychiatric interview for DSM-IV and ICD-10. J Clin Psychiatry. 1998;59:22-33.

17. Spitzer RL, Kroenke K, Williams JB, Löwe B. A brief measure for assessing generalized anxiety disorder: the GAD-7. Arch Intern Med. 2006;166(10):1092-7.

18. Tennant R, Hiller L, Fishwick R, Platt S, Joseph S, Weich S, et al. The Warwick-Edinburgh Mental Well-being Scale (WEMWBS): development and UK validation. Health Qual Life Outcomes. 2007;5:63.

19. Fresco DM, Moore MT, van Dulmen MH, Segal ZV, Ma SH, Teasdale JD, et al. Initial psychometric properties of the experiences questionnaire: validation of a self-report measure of decentering. Behav Ther. 2007;38(3):234-46.

20. Baer RA, Carmody J, Hunsinger M. Weekly change in mindfulness and perceived stress in a mindfulness-based stress reduction program. J Clin Psychol. 2012;68(7):755-65.

21. IAPT. Measuring recovery in IAPT services. 2014. Available at [10/11/2022]: http://www.oxfordahsn.org/wp-content/uploads/2015/11/measuring-recovery-2014.pdf

Appendix

A1 Adherence to MBCT practice

Adherence to home practice is captured with standard worksheets for MBCT. In line with common practice, we will distinguish between adherence to formal practice, i.e. meditation practices that follow recorded guidance and represent the main part of the daily homework for each week, and informal practice, i. e. smaller unguided practices that participants engage in to practice mindfulness in their daily lives. We will establish the percentage adherence to formal and informal practices in each week and derive indices of adherence to formal and informal practice by averaging over percentages across the 7 weeks of the course during which participants are asked to engage in practice. We will treat missing data as an indication that participants have not practiced and count any missing observations as 0, with the exception of cases where missing observations are clearly clustered in certain courses. The latter takes into account the fact that MBCT teachers are not always reliable in collecting home practice record sheets. If data indicate that missing values are clustered in particular courses, i.e. below 10% of data, we will exclude data from this course and treat them as missing rather than an indication that participants have not practiced.

A2 Example tables

[...]

C. Health Economics Analysis Plan (HEAP)

King's Health Economics
Health Economics Analysis Plan (HEAP) for a randomised controlled trial to investigate the clinical effectiveness and cost-effectiveness of Mindfulness-Based Cognitive Therapy (MBCT) for depressed non-responders to Increasing Access to Psychological Therapies (IAPT) high-intensity therapies

Acknowledgement: This HEAP is adapted from Supplementary material 1. Health Economics Analysis Plan (HEAP) template v1.0 in Jo Thorn et al Value in Health 24, ISSUE 4, P539-547, APRIL 01, 2021 Content of Health Economics Analysis Plans (HEAPs) for Trial-Based Economic Evaluations: Expert Delphi Consensus Survey

1.1 Title

Health Economics Analysis Plan (HEAP) for a randomised controlled trial to investigate the clinical effectiveness and cost-effectiveness of Mindfulness-Based Cognitive Therapy (MBCT) for depressed non-responders to Increasing Access to Psychological Therapies (IAPT) high-intensity therapies

1.2 Trial registration number

ISRCTN: ISRCTN17755571

1.3 Source of funding

National Institute for Health Research NIHR200750

1.4 Purpose of HEAP

The purpose of this HEAP is to describe the analysis and reporting procedure intended for the economic analyses to be undertaken. The analysis plan is designed to ensure that it is consistent and there is no conflict with the protocol and associated statistical analysis plan and it should be read in conjunction with them.

1.5 Trial protocol version

Version 3.0 [28.10.2022]

1.6 Trial Statistical Analysis Plan (SAP) version

SAP v1 21/04/2023

1.7 Trial HEAP version

Version 1.0

1.8 HEAP revisions

None

1.9 Roles and responsibilities

This HEAP was prepared by Dr Barbara Barrett (senior health economist). The trial health economists Dr Barbara Barrett and Poushali Ganguli are responsible for conducting and reporting the economic evaluation in accordance with the HEAP.

1.10 Signatures

Signature(s) of the person(s) writing the HEAP (and date)	Barbara Barrett 28/06/2023
Signature of senior health economist who is guarantor of the economic evaluation (and date)	Barbara Barrett 28/06/2023
Signature of the Chief Investigator for the trial (and date)	Thorsten Barnhofer 28/06/2023

1.11 Abbreviations/glossary of terms/definitions

Increasing Access to Psychological Therapies (IAPT)
Mindfulness-Based Cognitive Therapy (MBCT)
Treatment as Usual (TAU)
National institute for Health and Care Excellence (NICE).
Statistical Analysis Plan (SAP)
Health Economic Analysis Plan (HEAP)
Randomised Controlled Trial (RCT)
Patient Health Questionnaire – 9 (PHQ-9)
Quality-Adjusted Life Year (QALY)
Adult Service Use Schedule (AD-SUS)
EuroQol 5 Dimensions (EQ-5D-5L)

2.1 Trial background and rationale

Despite the success of the Increasing Access to Psychological Therapies (IAPT) services in terms of coverage and reach, outcome data suggest that around half of patients who receive high-intensity therapy do not fully recover [1]. There is evidence that Mindfulness-Based Cognitive Therapy (MBCT) is effective in people with persistent depression and those who have not responded to other psychological interventions [2, 3]. Evidence from a definitive clinical trial is needed to see the effectiveness and cost-effectiveness of MBCT for IAPT high-intensity treatment non-responders. As is appropriate for an economic evaluation, the comparator was Treatment as Usual (TAU).

2.2 Clearly and briefly state the main aim(s) of the trial

To establish
(a) the clinical effectiveness (in terms of reductions in depressive symptomatology) and (b) cost-effectiveness of MBCT as a psychotherapeutic treatment option for depressed patients who have not responded sufficiently to high-intensity evidence-based treatments within the IAPT care pathway compared with TAU.

2.3 Objectives and/or research hypotheses of the trial

(a) To undertake a definitive randomised controlled trial (RCT) of the MBCT intervention versus TAU to confirm clinical effectiveness of the treatment in depressed non-responders to high-intensity evidence-based treatments within the IAPT care pathway, and (b) To use the data from the RCT to conduct a cost-utility and cost-effectiveness analysis to provide information on whether or not the MBCT intervention is worthwhile in terms of improvements in outcomes or savings elsewhere in the health system.

2.4 Trial population

Patients aged 18 or older who have not responded to high-intensity IAPT interventions for depression (PHQ-9 score≥10 after 12 sessions), but do not meet eligibility criteria for secondary care services

2.5 Intervention(s) and comparator(s)

Intervention: MBCT (full details in the SAP)

Control: TAU (captured in the service use questionnaire)

2.6 Trial design

Individual randomised trial of 234 participants in 1:1 ratio to receive either MBCT or to continue with TAU. Full details in SAP.

2.7 Trial start and end dates

01/01/2021 – 31/12/2022

3.1 Aim(s) of economic evaluation

To establish the cost-effectiveness of MBCT as a psychotherapeutic treatment option for depressed patients who have not responded sufficiently to high-intensity evidence-based treatments within the IAPT care pathway compared with TAU.

3.2 Objective(s) of economic evaluation

(a) to estimate the cost of MBCT as delivered via videoconferencing, (b) to capture the range of services that constitute TAU (c) to conduct a cost-utility and cost-effectiveness analysis to provide information on whether or not the MBCT intervention is worthwhile in terms of improvements in outcomes or savings elsewhere in the health system.

3.3 Overview of economic analysis

Costs will be calculated by collecting service use information using the Adult Service Use Schedule (AD-SUS), a self-report measure developed by the team at King's College and used in previous trials of MBCT [5], modified for use online, to which routine unit costs will be applied [6]. We will collect data on all service use not just use related to mental health conditions, because there is evidence that successful treatment in IAPT can reduce use of all healthcare services [30]. In addition, comparison via randomised groups will ensure that any differences in cost are due to the impact of the MBCT intervention. Information on TAU will be collected via the AD-SUS, modified to ensure that all relevant services are included. Data on the use of the MBCT intervention will be collected via therapist records and costs estimated using the standard approach set out by Jones [6], acknowledging the challenges of costing group-based interventions [7]. Outcomes for the economic evaluation will be QALYs, calculated using health utilities derived from the EQ-5D-5L [8, 9]. Costs and

outcomes will be combined first in a cost-utility analysis using QALYS and second in a cost-effectiveness analysis using the PHQ-9, providing information on whether or not MBCT is worthwhile in terms of cost savings elsewhere or improvements in outcomes, and information will be provided to decision makers with statistical analysis of differences in costs, cost-effectiveness planes and cost-effectiveness acceptability curves [10]. Other approaches to represent the uncertainty in decision making will be considered once the data have been analysed.

3.4 Jurisdiction(s)

England, NHS services

3.5 Perspective(s)

The economic evaluation will take a health and social care perspective, as required for evidence presented to NICE. In addition, the cost perspective will be broadened to include the costs of time off productivity losses, since these are known to be relevant and important in those attending IAPT services [4].

3.6 Time horizon(s)

34 weeks

4.1 Statistical software

STATA 17 or higher

4.2 Identification of resources

We used previous research in major depression treatment to identify relevant items of service use [5].

4.3 Measurement of resource-use data

Adult Service Use Schedule (AD-SUS), a self-report measure developed by the team at King's College and used in previous trials of MBCT [5], modified for use online. At baseline, the questionnaire covers the previous 10 weeks, at 10 weeks follow-up the period since baseline and at 34 weeks follow-up the period since the 10 week follow-up.

4.4 Valuation of resource-use data, valuation of intervention resource-use

For each service use item unit costs will be applied using routine sources [6].

Data on the use of the MBCT intervention will be collected via therapist records and costs estimated using the standard approach set out by Jones [6], acknowledging the challenges of costing group-based interventions [7].

Discounting is not necessary as costs nor outcomes are not measured over 12 months.

4.5 Identification of outcome(s)

PHQ-9 scores at 34-week follow-up (consistent with end-of-treatment monitoring in IAPT).

4.6 Measurement of outcome(s)

Outcomes for the economic evaluation will be QALYs, calculated using health utilities derived from the EQ-5D-5L [8, 9].

4.7 Valuation of outcome(s)

Utility scores for the EQ-5D-5L will be applied according to the latest guidance from National Institute for Health and Care Excellence (NICE).

4.8 Monitoring collection of health economic data

Data for the economic evaluation will be monitored in collaboration with ExeCTU.

4.9 Database management, Data entry, Data archiving

Data from the online assessments are transferred directly into a secure, web-based system maintained by the ExeCTU and will be quality checked at the CTU. Consent forms will be stored separately from data and data will be anonymised wherever possible.

4.10 Data availability

Data analysis will be carried out on the final locked dataset.

5.1 Analysis population

Intention to treat analysis will be carried out.

5.2 Timing of analyses

Economic evaluation will take place at 34 weeks follow-up.

5.3 Discount rates for costs and benefits

Discounting of costs and benefits is not relevant since follow-up is at 34 weeks.

5.4 Cost-effectiveness threshold(s)

The primary economic analysis will use a cost-effectiveness threshold of £20,000 per QALY. (NICE cost-effectiveness threshold). The impact of uncertainty will be reported as the cost-effectiveness acceptability curve across a range of values for a QALY from zero to £50,000.

5.5 Statistical decision rule(s)

Mean differences in costs, QALYs and net benefits between the treatment groups will be estimated with associated 95% confidence intervals.

5.6 Analysis of resource use

Differences in the use of services between randomised groups will be described but not compared statistically.

5.7 Analysis of costs

Differences in overall mean costs between the arms will be analysed using a regression model to adjust for covariates. The following covariates have been identified from the SAP: depression severity (PHQ-9 <19 versus ≥ 19), antidepressant use at baseline and recruitment site with the addition of baseline costs.

5.8 Analysis of outcomes

Differences in overall mean QALYs between the arms will be analysed using a regression model to adjust for covariates. The following covariates have been identified from the SAP: depression

severity (PHQ-9<19 versus ≥19), antidepressant use at baseline and recruitment site with the addition of baseline EQ-5D utility score.

5.9 Data cleaning for analysis

Face validity tests will be conducted on data (e.g., to identify misspelt text) and checked against the source documents. Corrections identified will be documented in the code. All data cleaning procedures will be written as Stata syntax.

5.10 Missing data

Trial data will be examined for any missing data. Multiple imputation methods will be used based on the assumption that the data is missing at random (MAR), running bootstraps from the imputed data.

5.11 Analysis of cost-effectiveness

Cost and QALY data will be combined to calculate an incremental cost-effectiveness ratio (ICER) of MBCT and net monetary benefit (NMB) statistic from the NHS and PSS perspective. The net monetary benefit for MBCT and TAU will be reported after valuing a QALY at £20,000.

5.12 Sampling uncertainty

The nonparametric bootstrapping approach of trial data will be used to determine the level of sampling uncertainty surrounding the costs and effectiveness estimates and summary cost-effectiveness measures. Cost-effectiveness acceptability curves (CEAC) will be generated to consider the probability of MBCT being cost-effective for willingness to pay values between £0 and £50,000.

5.13 Subgroup analyses or analysis of heterogeneity

None.

5.14 Sensitivity analyses

Several sensitivity analyses will be undertaken to explore uncertainties surrounding key parameters in the economic evaluation. The impact of missing data will be identified by assessing whether treatment adherence is associated with missing data by conducting a complete case analysis. Productivity costs will be included in the sensitivity analysis.

6.1 Reporting standards

CHEERS guidelines will be followed when reporting the health economic evaluation, in a format appropriate to stakeholders and policy makers.

6.2 Deviations from the HEAP

Any deviation from the HEAP will be described and justified in the final published report.

References

[1] NHS Digital (2023) Psychological Therapies: reports on the use of IAPT services, England, March 2023 Final including a report on the IAPT Employment Advisers pilot and Quarter 4 2022-23 data. London: Department of Health.

[2] Barnhofer T, Crane C, Hargus E, Amarasinghe M, Winder R, Williams JM. Mindfulness-based cognitive therapy as a treatment for chronic depression: A preliminary study. Behav Res Ther. 2009;47(5):366-73.

[3] Winnebeck E, Fissler M, Gärtner M, Chadwick P, Barnhofer T. Brief training in mindfulness meditation reduces symptoms in patients with a chronic or recurrent lifetime history of depression: A randomized controlled study. Behav Res Ther. 2017;99:124-30.

[4] Layard R. The case for psychological treatment centres. BMJ. 2006;332:1030.

[5] Kuyken W, Hayes R, Barrett B, Byng R, Dalgleish T, Kessler D, et al. Effectiveness and cost-effectiveness of mindfulness-based cognitive therapy compared with maintenance antidepressant treatment in the prevention of depressive relapse or recurrence (PREVENT): a randomised controlled trial. Lancet. 2015;386:63–73.

[6] Jones KC, Weatherly H, Birch S, Castelli A, Chalkley M, Dargan A, Forder JE, Gao J, Hinde S, Markham S, Ogunleye D, Premji S, Roland D (2023) Unit Costs of Health and Social Care 2022 Manual. Technical report. Personal Social Services Research Unit (University of Kent) & Centre for Health Economics (University of York), Kent, UK

[7] Barrett B, Byford S. The challenges of estimating the unit cost of group-based therapies. In: Curtis L, editor. Unit Costs Health Soc Care. University of Kent: Personal Social Services Research Unit; 2008.

[8] EuroQuol Research Foundation. EQ-5D-5L User Guide, 2019. 2019 [cited 2020 Apr 17]. Available from: https://euroqol.org/publications/user-guides

[9] Devlin N, Shah K, Feng Y, Mulhern B, Hout VB. Valuing health-related quality of life: an EQ-5D-5L value set for England. Off Health Econ. 2016;27:7–22.

[10] Fenwick E, O'Brien BJ, Briggs A. Cost-effectiveness acceptability curves – facts, fallacies and frequently asked questions. Health Econ. 2004;13:405–15.

D. Sensitivity Analyses

D1. Sensitivity Analysis Taking into Account NHS Talking Therapies Services Effects

To address the potential effects of clustering by service, we conducted mixed effect regression models for the primary and secondary (continuous and binary) outcomes with a random effect on service. Table D1.1 shows the results of these analyses for continuous outcomes. Table D1.2 provides p-values for the random effect. Table D1.3 shows results of analyses for binary outcomes.

Supplementary Table D1.1

Mixed effect regression models for continuous outcomes with random effect on service (ITT, observed data)

	**10 Week Follow-Up**			**34 Week Follow-Up**		
**Outcome Measure**	**Mean Difference**	**95% CI**	**p-value**	**Mean Difference**	**95% CI**	**p-value**
**PHQ-9**	-2·5038	(-3·6271, -1·3805)	<0·0001	-2·4945	(-3·8672, -1·1217)	0·0004
**GAD-7**	-2·0659	(-3·0454, -1·0863)	<0·0001	-1·7223	(-2·9270, -0·5177)	0·0051
**Phobia Scale**	-0·0897	(-0·9566, 0·7773)	0·84	-0·6056	(-1·5958, 0·3847)	0·23
**WSAS**	-1·4939	(-3·0445, 0·0567)	0·059	-1·9592	(-3·7832, -0·1352)	0·035
**WEMWBS**	3·3203	(1·8065, 4·8341)	<0·0001	3·2472	(1·4007, 5·0937)	0·0006
**EQ-Decentering**	3·4613	(2·2478, 4·6747)	<0·0001	3·0714	(1·6373, 4·5056)	<0·0001
**FFMQ**	5·9258	(4·5075, 7·3442)	<0·0001	4·6127	(2·7508, 6·4746)	<0·0001

*PHQ-9 analyses adjusted for recruitment site, antidepressant medication use and baseline score. All other analyses adjusted for recruitment site, antidepressant medication use, depression severity (PHQ-9<19 versus ≥19) and baseline score.

Supplementary Table D1.2

Mixed effect regression models for continuous outcomes with random effect on service - p-values for the NHS-TT services effects

**Measure**	**NHS TT Effect p-value (10 weeks)**	**NHS TT Effect p-value (34 weeks)**
**PHQ-9**	1·00	1·00
**GAD-7**	1·00	1·00
**Phobia Scale**	0·237	1·00
**WSAS**	1·00	1·00
**WEMWBS**	1·00	1·00
**EQ-Decentering**	1·00	1·00
**FFMQ**	1·00	1·00

Supplementary Table D1.3

Summary of mixed-effect logistic regression models, adjusting for NHS TT service (ratios using full number of participants entering treatment arm as denominator)

	% [n/N]		OR [95% CI] [a]		*p* [a]		*NHS TT Effect p-value*	
**Outcome/ Group**	**10-week**	**34-week**	**10-week**	**34-week**	**10-week**	**34-week**	**10-week**	**34-week**
**PHQ-9 only**								
Recovery			2·25 [1·01, 5·01]	2·09 [1·09, 4·01]	·047	·026	·	·
MBCT	18 [22/118]	27 [33/118]						
TAU	9 [11/116]	15 [18/116]						
Reliable Improvement			3·03 [1·55, 5·92]	1·82 [1·03, 3·23]	·0012	·039	·	·
MBCT	31 [37/118]	38 [46/118]						
TAU	13 [16/116]	26 [31/116]						
Reliable Recovery			3·69 [1·40, 9·75]	2·33 [1·13, 4·80]	·0085	·021	·	·
MBCT	16 [19/118]	22 [27/118]						
TAU	5 [6/116]	11 [13/116]						
Reliable Deterioration			0·34 [0·03, 4·36]	0·98 [0·13, 7·34]	·41	·98	·	·
MBCT	0 [1/118]	1 [2/118]						
TAU	1 [2/116]	1 [2/116]						
**GAD-7 only**								
Reliable Improvement			3·43 [1·79, 6·60]	2·00 [1·12, 3·56]	·0002	·019	·	·
MBCT	35 [42/118]	37 [44/118]						
TAU	14 [17/116]	23 [27/116]						
Reliable Deterioration			0·75 [0·30, 1·91]	0·96 [0·40, 2·27]	·55	·92	·	·
MBCT	7 [9/118]	10 [12/118]						
TAU	9 [11/116]	11 [13/116]						
**PHQ-9/GAD-7**								
Recovery			4·64 [1·43, 15·00]	1·83 [0·85, 3·93]	·010	·12	·	·
MBCT	12 [15/118]	17 [21/118]						
TAU	3 [4/116]	20 [24/116]						
Reliable Improvement			2·67 [1·49, 4·77]	1·98 [1·16, 3·40]	·0009	·013	·	·
MBCT	42 [50/118]	49 [58/118]						
TAU	22 [26/116]	36 [42/116]						
Reliable Recovery			8.91[1·92, 41·33]	2·25 [0·97, 5·21]	·0052	·059	·	·
MBCT	22 [14/118]	27 [19/118]						
TAU	6 [2/116]	16 [9/116]						
Reliable Deterioration			0·58 [0·22, 1·50]	1·15 [0·47, 2·80]	·26	·76	·	·
MBCT	6 [8/118]	10 [12/118]						
TAU	10 [12/116]	9 [11/116]						

[a] Adjusted for depression severity (PHQ-9 score <19 vs ≥19), antidepressant use at baseline, and recruitment site

[*] Where p-value is recorded as '.', this is a Chi-Square statistic equal to 0, so no p-value is derived.

D2. Sensitivity Analysis Taking into Account Therapist Effects

Potential effects of clustering by therapist were investigated using a mixed effects linear regression models for the primary and secondary (continuous) outcomes with a random effect on therapist within the MBCT group only. P values for the random effect from analyses are listed in table D2.1. In addition, we performed mixed effects linear regression models for continuous outcomes with a random effect on therapist and therapist experience as a parameter within the model. P values of these analyses are listed in table D2.2.

Supplementary Table D2.1

Single-arm linear models (MBCT group only) with therapist effect adjusted for as a random effect (ITT, observed data)

Measure	Therapist Effect p-value (10 weeks)	Therapist Effect p-value (34 weeks)
**PHQ-9**	0·11	1·00
**GAD-7**	1·00	1·00
**Phobia Scale**	0·19	1·00
**WSAS**	1·00	1·00
**WEMWBS**	1·00	1·00
**Decentering Scale**	1·00	1·00
**FFMQ**	1·00	1·00

Supplementary Table D2.2

Single-arm linear models (MBCT group only) with therapist effect adjusted for as a random effect and including therapist experience as a parameter within the model (ITT, observed data)

Measure	Therapist Years Teaching MBCT p-value (10 Weeks)	Therapist Years Teaching MBCT p-value (34 Weeks)	Therapist Years since Qualification p-value (10 Weeks)	Therapist Years since Qualification p-value (10 Weeks)
**PHQ-9**	0·091	0·99	0·48	0·22
**GAD-7**	0·062	0·23	0·23	0·26
**Phobia Scale**	0·16	0·51	0·30	0·34
**WSAS**	0·86	0·20	0·066	0·18
**WEMWBS**	0·11	0·99	0·81	0·26
**Decentering Scale**	0·63	0·61	0·67	0·45
**FFMQ**	0·44	0·59	0·91	0·84

D3. Sensitivity Analysis Taking into Account Different Inclusion Criteria

To explore effects under conditions of different inclusion criteria, we performed a sensitivity analysis excluding participants who had shown reliable improvement in PHQ-9 (i.e. a reduction by 6 points on more on PHQ-9) during NHS Talking Therapies treatment, thus focussing on participants who in addition to having failed to reach recovery had also failed to show reliable improvement over the course of their previous treatment (84 of 118 participants in the MBCT arm and 79 of the 116 participants in the TAU arm). This sensitivity analysis was performed using regression modelling for the primary and secondary outcomes. Results for continuous outcomes are listed in table D3.1, results for binary outcomes in table D3.2.

Supplementary Table D3.1

Linear regression models for continuous outcomes excluding participants who showed reliable improvement in NHS TT high-intensity therapy (ITT, observed data)

	**10 Week Follow-Up**			**34 Week Follow-Up**		
**Outcome Measure**	**Mean Difference**	**95% CI**	**p-value**	**Mean Difference**	**95% CI**	**p-value**
**PHQ-9**	-1·8396	(-3·1455, -0·5337)	0·0061	-2·5866	(-4·2255, -0·9477)	0·0022
**GAD-7**	-1·9347	(-3·1083, -0·7611)	0·0014	-2·0073	(-3·4657, -0·5488)	0·0073
**Phobia Scale**	0·2861	(-0·7416, 1·3137)	0·58	-0·1344	(-1·3449, 1·0760)	0·83
**WSAS**	-1·0947	(-2·9282, 0·7388)	0·24	-1·7132	(-3·9165, 0·4901)	0·13
**WEMWBS**	2·3374	(0·6065, 4·0683)	0·0085	2·9658	(0·8259, 5·1057)	0·0070
**EQ-Decentering**	2·6197	(1·2273, 4·0121)	0·0003	2·2627	(0·5222, 4·0033)	0·011
**FFMQ**	5·8779	(4·1273, 7·6285)	<0·0001	3·5362	(1·3009, 5·7715)	0·0022

*PHQ-9 analyses adjusted for recruitment site, antidepressant medication use and baseline score. All other analyses adjusted for recruitment site, antidepressant medication use, depression severity (PHQ-9<19 versus ≥19) and baseline score.

Supplementary Table D3.2

Summary of logistic regression models, excluding participants who showed reliable improvement in previous NHS TT high-intensity therapy (ratios using full number of participants entering treatment arm as denominator)

	**% [n/N]**		**OR [95% CI] [a]**		***p* [a]**	
**Outcome/ Group**	**10-week**	**34-week**	**10-week**	**34-week**	**10-week**	**34-week**
**PHQ-9 only**						
Recovery			1·28 [0·50, 3·29]	2·09 [0·95, 4·59]	·60	·068
MBCT	15 [12/84]	30 [23/84]				
TAU	13 [10/79]	19 [13/79]				
Reliable Improvement			2·41 [1·10, 5·29]	1.72 [0·87, 3·41]	·028	·12
MBCT	33 [26/84]	45 [34/84]				
TAU	16 [12/79]	32 [22/79]				
Reliable Recovery			2·02 [0·69, 5·93]	2·18 [0·90, 5·23]	·20	·077
MBCT	13 [11/84]	21 [18/84]				
TAU	7 [6/79]	11 [9/79]				
Reliable Deterioration			··	1·29 [0·07, 22·42]	··	·86
MBCT	0 [0/84]	1 [1/84]				
TAU	0 [0/79]	1 [1/79]				
**GAD-7 only**						
Reliable Improvement			3·44 [1·52, 7·35]	2·74 [1·33, 5·62]	·003	·006
MBCT	35 [30/84]	41 [35/84]				
TAU	15 [12/79]	20 [16/79]				
Reliable Deterioration			0·60 [0·20, 1·83]	0·71 [0·25, 2·03]	·371	·523
MBCT	7 [6/84]	10 [8/84]				
TAU	12 [9/79]	14 [10/79]				
**PHQ-9/GAD-7**						
Recovery			3·38 [0·93, 12·25]	1·64 [0·63, 4·25]	·063	·31
MBCT	11 [10/84]	15 [13/84]				
TAU	5 [4/79]	10 [8/79]				
Reliable Improvement			2·42 [1·21, 4·86]	2·49 [1·28, 4·85]	·012	·0070
MBCT	42 [36/84]	54 [46/84]				
TAU	24 [19/79]	32 [26/79]				
Reliable Recovery			7·21 [1·42, 36·74]	2·05 [0·72, 5·81]	·017	·18
MBCT	11 [10/84]	14 [12/84]				
TAU	2 [2/79]	7 [6/79]				
Reliable Deterioration			0·60 [0·20, 1·83]	0·97 [0·32, 2·91]	·37	·95
MBCT	7 [6/84]	9 [8/84]				
TAU	11 [9/79]	10 [8/79]				

[a] Adjusted for depression severity (PHQ-9 score <19 vs ≥19), antidepressant use at baseline, and recruitment site

D4. Sensitivity Analysis Taking into Account Size of Assessment Window

We performed a sensitivity analysis for the primary continuous outcomes to include data collected during the first 7-days of the data collection window only. Results of these analyses are listed in Table D4.1. Figure D4.1 shows the distribution of days taken to complete assessments at final follow-up. In the MBCT group, the average number of days until completion was *M* = 10·66 (*SD* = 10·94, *Med* = 8, *range* = [1, 47]), in the TAU group the average number of days until completion was *M* = 9·78 (*SD* = 10·94, *Med* = 6, *range* = [1, 57]).

Supplementary Table D4.1

Linear regression models for continuous outcomes excluding responses obtained out of initial 7-day window (ITT, observed data)

	**34 Week Follow-Up**		
**Outcome Measure**	**Mean Difference**	**95% CI**	**p-value**
**PHQ-9**	-3·1415	(-5·0342, -1·2488)	0·0014
**GAD-7**	-2·1065	(-3·6762, -0·5369)	0·0091
**Phobia Scale**	-1·0857	(-2·4575, 0·2860)	0·12
**WSAS**	-3·6119	(-6·1937, -1·0302)	0·0066
**WEMWBS**	3·3861	(0·9445, 5·8277)	0·0071
**EQ-Decentering**	2·8764	(0·7022, 5·0506)	0·010
**FFMQ**	4·9312	(1·8118, 8·0508)	0·0023

*PHQ-9 analyses adjusted for recruitment site, antidepressant medication use and baseline score. All other analyses adjusted for recruitment site, antidepressant medication use, depression severity (PHQ-9<19 versus ≥19) and baseline score.

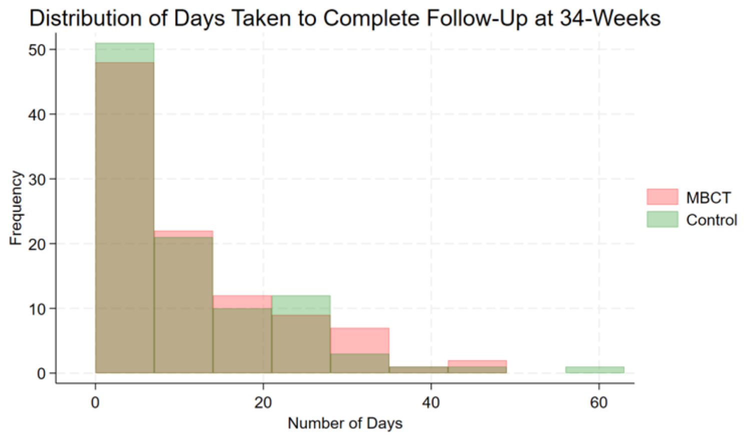

Supplementary Figure D4.1

Distribution of Days Taken to Complete Follow-Up at Final Follow-Up

D5. Complier Average Causal Effect Analysis

A CACE analysis was performed, as a further sensitivity analysis, to estimate the treatment effect while accounting for non-adherence to treatment. A participant in the intervention arm was considered to be a ‘complier’, if a minimum of four treatment sessions were attended. A 2-stage least squared instrumental variable regression model was used for the purpose of identifying those participants in the TAU group who would be ‘compliers’ had they been allocated to MBCT and comparing the compliers in both groups. A CACE analysis was performed for the PHQ-9 and all continuous secondary outcomes. Table D5.1 shows the outcomes of this analysis.

Supplementary Table D5.1

CACE Analysis (Observed Data, Eligible Participants Only)

	**10 Week Follow-Up**			**34 Week Follow-Up**		
**Outcome Measure**	**Mean Difference**	**95% CI**	**p-value**	**Mean Difference**	**95% CI**	**p-value**
**PHQ-9**	-2·7679	(-4·0125, -1·5234)	<0·0001	-2·8081	(-4·3384, -1·2779)	0·0003
**GAD-7**	-2·2776	(-3·3636, -1·1916)	<0·0001	-1·9321	(-3·2753, -0·5889)	0·0048
**Phobia Scale**	-0·1003	(-1·0637, 0·8630)	0·84	-0·6818	(-1·7966, -0·4330)	0·23
**WSAS**	-1·6451	(-3·3532, 0·0630)	0·059	-2·2009	(-4·2492, -0·1526)	0·035
**WEMWBS**	3·6710	(1·9910, 5·3510)	<0·0001	3·6527	(1·5715, 5·7338)	0·0006
**EQ-Decentering**	3·8283	(2·5120, 5·1446)	<0·0001	3·4553	(1·8808, 5·0298)	<0·0001
**FFMQ**	6·5659	(5·0393, 8·0925)	<0·0001	5·1980	(3·1571, 7·2388)	<0·0001

*PHQ-9 analyses adjusted for recruitment site, antidepressant medication use and baseline score. All other analyses adjusted for recruitment site, antidepressant medication use, depression severity (PHQ-9<19 versus ≥19) and baseline score.

D6. Sensitivity Analyses with Imputed Missing Data

Observed and imputed data were used to perform a sensitivity analysis of the inferential between group comparisons at 34 weeks. Results are presented in Table D6.1. Missing outcome data for continuous outcome measures (PHQ-9, GAD-7, Phobia Scale, WSAS, WEMWBS, FFMQSF, EQ Decentering Score, at 10 weeks and 34 weeks) was imputed using multiple imputation with chained equations with predictive mean matching to impute missing observations. Based on the percentage of participants (out of 234) with at least one missing outcome observation at 10 weeks or 34 weeks, 17 datasets were imputed. Imputed models included treatment group, baseline scores for the outcome measures, and factors used in randomisation (site and antidepressant use, but not binarised PHQ-9 score, as continuous PHQ-9 score at baseline was included) to

impute the missing data. In addition, demographic variables (age, gender, marital status, ethnicity, highest educational attainment, annual household income, age of onset of depression, number of previous depressive episodes) were assessed for association with the primary outcome (PHQ-9 at 34 weeks) and missingness of the primary outcome. In a linear regression model with adjustment for treatment group, site, antidepressant use and baseline PHQ-9 score, there was weak evidence for an association between age and PHQ-9 at 34 weeks (p-value 0·059). Similarly, in a logistic regression model for missingness of primary outcome data, adjusting for treatment group, site and baseline depression severity (binarised PHQ-9 score), age and age of onset were found to be predictive of missingness of the primary outcome (p-values 0·048 and 0·013 respectively). Therefore, age and age of onset were added as auxiliary variables into the imputation model.

Supplementary Table D6.1

Intention to treat analysis (observed and imputed data)

	**10 Week Follow-Up**			**34 Week Follow-Up**		
**Outcome Measure**	**Mean Difference**	**95% CI**	**p-value**	**Mean Difference**	**95% CI**	**p-value**
**PHQ-9**	-2·36	(-3·49, -1·23)	<0·001	-2·38	(-3·75, -1·01)	0·0008
**GAD-7**	-1·99	(-2·97, -1·01)	<0·001	-1·60	(-2·81, -0·40)	0·0093
**Phobia Scale**	-0·04	(-0·93, 0·85)	0·937	-0·38	(-1·42, 0·65)	0·47
**WSAS**	-1·47	(-3·03, 0·09)	0·064	-1·68	(-3·49, 0·12)	0·067
**WEMWBS**	3·25	(1·69, 4·82)	<0·001	3·19	(1·33, 5·06)	0·0009
**EQ-Decentering**	3·45	(2·24, 4·65)	<0·001	2·91	(1·47, 4·35)	0·0001
**FFMQ**	5·68	(4·27, 7·08)	<0·001	4·54	(2·68, 6·40)	<0·0001

*PHQ-9 analyses adjusted for recruitment site, antidepressant medication use and baseline score. All other analyses adjusted for recruitment site, antidepressant medication use, depression severity (PHQ-9<19 versus ≥19) and baseline score.

D7. Repeated Measures Analysis

A repeated measures analysis was performed for the primary outcome and continuous secondary outcomes, using a mixed effects linear regression model with a random effect on participant (i.e. observations at different timepoints will be nested within participant), and including an interaction term between treatment group and timepoint. All other predictors were included as fixed effects within the model. This model included participants with follow-up data reported for at least one follow-up time, using observed data according to the ITT principle. Table D7.1 shows the main outcomes of this analysis.

Supplementary Table D7.1

Repeated measures analysis (analysis conducted with interaction between treatment and timepoint using 10-week timepoint and control group as reference)

	**34 Week Follow-Up**		
**Outcome Measure**	**Mean Difference**	**95% CI**	**p-value**
PHQ-9	-2·4618	(-3·6912, -1·2324)	0·0001
GAD-7	-2·0303	(-3·1098, -0·9508)	<0·0002
Phobia Scale	-0·01287	(-1·0526, 0·7953)	0·78
WSAS	-1·5101	(-3·1714, 0·1512)	0·075
WEMWBS	3·2728	(1·6189, 4·9268)	0·0001
EQ-Decentering	3·4971	(2·1959, 4·7982)	<0·0001
FFMQ	5·8791	(4·2644, 7·4938)	<0·0001

*PHQ-9 analyses adjusted for recruitment site, antidepressant medication use and baseline score. All other analyses adjusted for recruitment site, antidepressant medication use, depression severity (PHQ-9<19 versus ≥19) and baseline score.

D8. Sensitivity Analysis to Handle Post-Randomisation Ineligible Participants

The trial included 25 participants who were randomised in error and therefore excluded from the primary analysis. As a further sensitivity analysis, we ran the primary analysis with these participants included. Descriptives are listed in table D8.1, main results of the linear regression model are listed in table D8.2.

Supplementary Table D8.1

Descriptive statistics for PHQ-9 for all randomised participants (ITT analysis, observed data)

	**Baseline**		**10 Week Follow-Up**		**34 Week Follow-Up**	
	**MBCT** (N=129)	**TAU** (N=130)	**MBCT** (N=114)	**TAU** (N=121)	**MBCT** (N=108)	**TAU** (N=114)
**PHQ-9, Mean (SD)**	17·65 (4·03)	17·58 (3·78)	13·63 (5·37)	15·64 (4·91)	12·41 (5·52)	14·24 (5·72)

Supplementary Table D8.2

Linear regression model for PHQ-9 for all randomised participants (ITT analysis, observed data)

	10 Week Follow-Up			34 Week Follow-Up		
	**Mean difference**	**95% CI**	**p-value**	**Mean difference**	**95% CI**	**p-value**
**PHQ-9**	-2·3806	(-3·4953, -1·2659)	<0·001	-2·1169	(-3·4698, -0·7641)	0·0023

D9. Rates of Participants Showing at least 50% Reduction of Depressive Symptomatology (PHQ-9) in MBCT+TAU and TAU at 10-weeks and 34-weeks

Supplementary Table D9

Rates of Participants Showing at least 50% Reduction of Depressive Symptomatology (PHQ-9) in MBCT+TAU and TAU at 10-weeks and 34-weeks

			OR [95% CI] [a]		*p* [a]	
**Outcome/ Group**	**10-week**	**34-week**	**10-week**	**34-week**	**10-week**	**34-week**
PHQ-9			3·20 [1·21, 8·50]	1·67 [0·84, 3·31]	·020	·14
MBCT+TAU	14 [17/118]	22 [26/118]				
TAU	5 [6/116]	14 [17/116]				

Ratios use the full number of participants entering the treatment arm at randomisation as denominator, which means that unless questionnaire data explicitly demonstrate response patients were counted as non-responders including those for whom no further data are available.

Number needed to treat (NNT) based on 50% reduction was 12 at 10 weeks and 13 at 34 weeks.

D10. Summary of Logistic Regression Models Predicting Dichotomous Outcomes at 10-week and 34-week Follow-Up using Baseline Scores as Covariates

Supplementary Table D10

Summary of logistic regression models predicting dichotomous outcomes at 10-week and 34-week follow-up using baseline scores as covariates

			**OR [95% CI] [a]**		***p* [a]**	
**Outcome/ Group**	**10-week**	**34-week**	**10-week**	**34-week**	**10-week**	**34-week**
**PHQ-9 only**						
Recovery *			2·42 [1·08, 5·44]	2·20 [1·14, 4·23]	·033	·019
MBCT+TAU	18 [22/118]	27 [33/118]				
TAU	9 [11/116]	15 [18/116]				
Reliable Improvement *			3·00 [1·53, 5·89]	1·78 [1·00, 3·17]	·0014	·049
MBCT+TAU	31 [37/118]	38 [46/118]				
TAU	13 [16/116]	26 [31/116]				
Reliable Recovery *			3·74 [1·42, 9·85]	2·35 [1·15, 4·84]	·0076	·020
MBCT+TAU	16 [19/118]	22 [27/118]				
TAU	5 [6/116]	11 [13/116]				
Deterioration *			0·38 [0·19, 0·74]	0·16 [0·06, 0·40]	·0046	·0001
MBCT+TAU	14 [17/118]	5 [6/118]				
TAU	30 [35/116]	25 [29/116]				
Reliable Deterioration *			0·37 [0·02, 5·99]	1·00 [0·13, 7·56]	·48	1·00
MBCT+TAU	0 [1/118]	1 [2/118]				
TAU	1 [2/116]	1 [2/116]				
**GAD-7 only**						
Deterioration **			0.37 [0.19, 0.72]	0.56 [0.30, 1.03]	·0036	·063
MBCT+TAU	17 [21/118]	21 [25/118]				
TAU	34 [40/116]	32 [38/116]				
Reliable Deterioration **			0·74 [0·27, 2.04]	1·08 [0·43, 2·72]	·56	·87
MBCT+TAU	7 [9/118]	10 [12/118]				
TAU	9 [11/116]	11 [13/116]				
**PHQ-9/GAD-7**						
Recovery ***			5·12 [1·55, 16·93]	1·90 [0·88, 4·10]	·0074	·10
MBCT+TAU	12 [15/118]	17 [21/118]				
TAU	3 [4/116]	10 [12/116]				
Reliable Improvement ***			2·68 [1·49, 4·81]	1.97 [1·14, 3·39]	·0010	·015
MBCT+TAU	42 [50/118]	49 [58/118]				
TAU	22 [26/116]	33 [39/116]				
Reliable Recovery ***			9·44 [2·02, 44·18]	2·26 [0·97, 5·26]	·0043	·057
MBCT+TAU	11 [14/118]	16 [19/118]				
TAU	1 [2/116]	7 [9/116]				
Reliable Deterioration ***			0·57 [0·21, 1.57]	1·26 [0·50, 3·20]	·28	·62
MBCT+TAU	6 [8/118]	10 [12/118]				
TAU	10 [12/116]	9 [11/116]				

Data are % and ratios (n/N) of participants meeting criteria for recovery, reliable improvement, reliable recovery, deterioration, and reliable deterioration. Ratios use the full number of participants entering the treatment arm at randomisation as denominator to align with the "demonstrated recovery" principle used for national NHS TT statistics,

which posits that unless questionnaire data explicitly demonstrate recovery, patients need to be counted as not having reached remission including those for whom no further data are available. It is important to note that not all participants reported anxiety levels at the level of caseness at baseline. [a] Logistic regression models for dichotomous outcomes based on PHQ-9 adjusted for baseline PHQ-9, antidepressant use at baseline, and recruitment site, but not depression severity (PHQ-9 score <19 vs ≥19) due to collinearity. Logistic regression models for dichotomous outcomes based on GAD-7 adjusted for baseline GAD-7, depression severity (PHQ-9 score <19 vs ≥19), antidepressant use at baseline, and recruitment site. Logistic regression models for dichotomous outcomes based on the combination of PHQ-9 and GAD-7 adjusted for baseline PHQ-9 and baseline GAD-7, antidepressant use at baseline, and recruitment site, but not depression severity (PHQ-9 score <19 vs ≥19) due to collinearity. [*] Recovery = reduction of depression symptoms to a level below caseness (PHQ-9 < 10), reliable improvement = reduction in depression symptom score greater than reliable change index (PHQ-9 ≥ 6), reliable recovery = reduction of depression symptoms to reach reliable improvement and recovery, deterioration = any increase in depression symptom score on the PHQ-9, reliable deterioration = increase in depression symptom score greater than reliable change index (PHQ-9 ≥ 6). [**] Deterioration = any increase in anxiety symptom score on the GAD-7, reliable deterioration = increase in anxiety symptom score greater than reliable change index (GAD-7 ≥ 4). [***] Recovery = reduction of depression and anxiety symptoms to a level below caseness (PHQ-9 < 10 and GAD-7 < 8), reliable improvement = reduction in depression symptom score and anxiety symptom score greater than reliable change index (PHQ-9 ≥ 6 and/or GAD-7 ≥ 4) or reduction in depression symptom score or anxiety symptom score without reliable deterioration on the other, reliable recovery = reliable improvement and recovery. MBCT+TAU = Mindfulness-Based Cognitive Therapy plus treatment as usual, TAU = treatment as usual.

D11. Linear Regression Models for PHQ-9 Adjusting for Relationship Status (Partner Yes/No) and Randomisation Variables (Depression Severity, Antidepressant Use at Baseline, Site)

Supplementary Table D11

Linear Regression Models for PHQ-9 Adjusting for Relationship Status (Partner Yes/No) and Randomisation Variables (Depression Severity, Antidepressant Use at Baseline, Site)

	**OR [95% CI] [a]**		***p* [a]**	
**Outcome**	**10-week**	**34-week**	**10-week**	**34-week**
PHQ-9 including Partner (Yes/No)	-2·53 [-3·70, -1·37]	-2·50 [-3·92, -1·08]	<·0001	·0007

D12. Line Graph of PHQ-9 Scores at Baseline, 10 Weeks and 34 Weeks

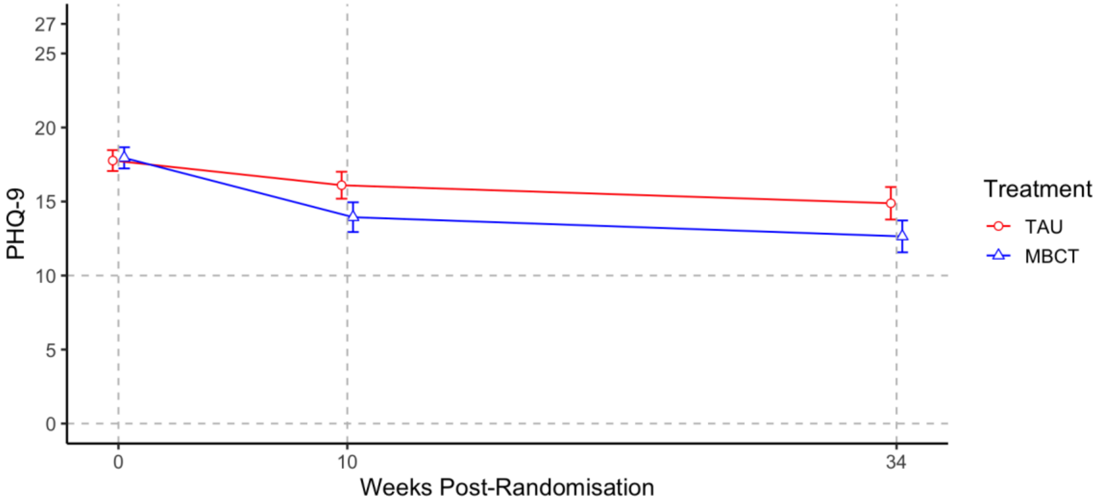

Figure D1

Means and 95% CIs of PHQ-9 at 0-, 10-, and 34-weeks post-randomisation

Circles and triangles indicate mean scores, upper and lower bars indicated 95% CIs. PHQ-9 = Patient Health Questionnaire-9, higher scores on the PHQ-9 indicate higher levels of symptoms. PHQ-9 at 34-week follow up represents the primary outcome of the trial. The gray dashed horizontal line crossing the y-axis at value 10 indicates the clinical cut-off on the PHQ-9, i.e. patients scoring 10 or more are considered to be at caseness.

E. Health Economic Analyses

E1. Unit Costs and Sources Used for the Economic Evaluation

Supplementary Table E1

Unit Costs and Sources Used for the Economic Evaluation

Service	Unit cost or range	Source	Notes
Hospital inpatient (per night/episode)	341 to 985	PSSRU, 2022	Mental health care clusters (per bed day); Non-elective inpatient stays (short stays)
Hospital outpatient (per appointment)	165 to 295	NHS Reference costs 2021/22	Average of all total outpatient attendances; total outpatient attendance tab, adult mental health service, service code 710
Hospital accident and emergency (per attendance)	144 to 390	NHS Reference costs 2021/22	Varied by if ambulance services were used
Community-based health and social care	12 to 239	NHS Reference costs 2021/22 & PSSRU, 2022	Varied by specialist seen
Medication (per week)	8·50	PCA, 2022	Per item, per month
Missed employment (per day)	32·57 to 103·35	ONS, 2022	Varied by part-time or full-time employment
MBCT intervention per person per session	10.12	Micro-costing using PSSRU, 2022	Assumes a band 7 therapist and a group size of 13. Health service costs only.

E2. Use of Health and Social Care Services

Supplementary Table E2

Use of health and social care services and missed employment over 34-weeks' follow-up

	MBCT (n=94)			TAU (n=95)		
**Service**	**Mean (SD)**	**Range**	**Percentage using**	**Mean (SD**	**Range**	**Percentage using**
**Hospital**						
Inpatient (nights)	0·19 (1·07)	0 to 9	4·26	0·13 (0·62)	0 to 5	6·32
Outpatient (apps)	1·65 (3·74)	0 to 21	36·17	2·70 (4·74)	0 to 32	57·89
A&E (attendances)	0·12 (0·32)	0 to 1	11·70	0·20 (0·54)	0 to 3	15·79
Ambulance (attendances)	0·02 (0·15)	0 to 1	2·13	0·04 (0·25)	0 to 2	3·16
**Community health and social care (contacts)**						
GP (surgery)	1·39 (2·03)	0 to 11	57·45	1·98 (2·64)	0 to 12	57·89
GP (home)	-*	-*	-*	0·03 (0·31)	0 to 3	1·05
GP (phone)	1·70 (2·25)	0 to 8	46·81	2·38 (2·79)	0 to 12	57·89
Nurse in a GP surgery	1·14 (1·76)	0 to 10	45·74	1·24 (2·28)	0 to 12	42·11
District nurse, health visitor or midwife	0·14 (0·92)	0 to 8	3·19	0·15 (1·05)	0 to 10	4·21
Community psychiatric nurse, key worker or home treatment team	0·05 (0·31)	0 to 2	3·19	0·11 (0·49)	0 to 3	5·26
Psychiatrist in the community	-*	-*	-*	0·15 (0·67)	0 to 5	6·32
Art/drama/music therapist in the community	0·11 (1·03)	0 to 10	1·06	0·06 (0·62)	0 to 6	1·05
Counsellor/clinical psychologist/therapist	1·85 (4·44)	0 to 24	26·60	2·66 (4·31)	0 to 17	40·0
Other service/professional in community	0·70 (2·12)	0 to 14	17·02	0·85 (3·26)	0 to 22	15·79
**Employment**			**Percentage missing work**			**Percentage missing work**
Days missed due to mental health	5·22 (22·57)	0 to 170	26·60	8·74 (21·14)	0 to 140	45·26

* Dash represents 0.

E3. Medication Use

Supplementary Table E3

Medication use over 34 weeks follow-up

	Trial group, percentage using	
**Medication type**	**MBCT (n = 94)**	**TAU (n = 95)**
Medication for depression	69·15	73·68
Medication for anxiety	52·13	58·95
Medication for psychosis	2·13	0
Medication for sleep problems	26·60	28·42

E4. Health and Social Care Perspective (Primary Analysis)

E4.1 Health and Social Care Perspective (Complete Case)

Supplementary Table E4.1.1

Mean costs (£) per participant over 34 weeks follow-up (health and social care perspective, complete case)

	Trial group, mean (SD)		MBCT minus TAU[a]			
**Cost category**	**MBCT**	**TAU**	**Unadjusted difference[b]**	**Adjusted difference[b,c]**	**95% CI**	**p-value**
**Baseline**						
Total	483·94 (780·10)	606·65 (1,021·43)	-122·70	-121·46	-387·93 to 145·01	0·37
**10-week follow-up**						
Hospital services	114·31 (290·85)	180·40 (395·31)	-66·08	-47·06	-146·86 to 52·73	0·36
Community-based services	113·76 (197·90)	128·19 (257·20)	-14·43	-10·72	-76·30 to 54·85	0·75
Medication	25·54 (22·81)	31·09 (22·53)	-5·55	-1·02	-5·34 to 3·30	0·64
Total	253·62 (410·66)	339·69 (538·01)	-86·06	-50·10	-182·98 to 82·77	0·46
**34-week follow-up**						
MBCT intervention	67·72 (20·02)	0 (0)	67·72	67·77	63·69 to 71·85	<0·0001
Hospital services	383·40 (830·49)	647·85 (1,042·67)	-264·46	-230·37	-497·04 to 36·30	0·090
Community-based services	340·93 (474·59)	457·09 (506·03)	-116·15	-112·29	-248·65 to 24·08	0·11
Medication	96·62 (73·24)	99·81 (72·25)	-3·18	11·06	-2·67 to 24·80	0·11
**Total**	**888·66 (1,132·22)**	**1,204·75 (1,322·28)**	**-316·09**	**-245·23**	**-581·92 to 91·46**	**0·15**

[a] Comparison based on complete case data, [b] Unadjusted and adjusted differences based on 5,000 bootstrapped replications, [c] Adjusted for depression severity (PHQ-9 score <19 vs ≥19), antidepressant use at baseline, recruitment site and baseline costs

Supplementary Table E4.1.2

Mean utility scores and QALYs per participant (health and social care perspective, complete case)

	Trial group, mean (SD)			MBCT minus TAU[a]		
**Quality of life**	**MBCT**	**Usual care**	**Unadjusted difference[b]**	**Adjusted difference[b,c]**	**95% CI**	**p-value**
Baseline (utility score)	0·524 (0·265)	0·512 (0·271)	0·012	0·009	-0·063 to 0·082	0·80
10 weeks (utility score)	0·559 (0·272)	0·491 (0·288)	0·068	0·062	0·009 to 0·115	0·022
34 weeks (utility score)	0·568 (0·289)	0·504 (0·310)	0·064	0·564	-0·004 to 0·117	0·069
34 weeks (QALYs)	0·364 (0·166)	0·326 (0·180)	0·038	0·033	0·005 to 0·061	0·020

[a] Comparison based on complete case data, [b] Unadjusted and adjusted differences based on 5,000 bootstrapped replications, [c] Adjusted for depression severity (PHQ-9 score <19 vs ≥19), antidepressant use at baseline, recruitment site and baseline utility

Supplementary Table E4.1.3

NHS direct delivery costs based on intervention cost and NHS TT binary outcomes at 10-week and 34-week follow up

	Direct delivery cost per person achieving outcome (in £)	
**Binary outcome**	**10-week FU**	**34-week FU**
PHQ-9 recovery*	363·22	242·15
PHQ-9 reliable improvement*	215·97	173·72
PHQ-9 reliable recovery*	420·57	295·96
PHQ-9/GAD-7 recovery**	532·73	380·52
PHQ-9/GAD-7 reliable improvement**	159·81	137·76
PHQ-9/GAD-7 reliable recovery**	570·78	420·57

Ratios for binary outcomes use the full number of participants entering the treatment arm at randomisation as denominator to align with the "demonstrated recovery" principle used for national NHS TT statistics, which posits that unless questionnaire data explicitly demonstrate recovery patients need to be counted as non-responders including those for whom no further data are available. * Recovery = reduction of depression symptoms to a level below caseness (PHQ-9 < 10), reliable improvement = reduction in depression symptom score greater than reliable change index (PHQ-9 ≥ 6), reliable recovery = reduction of depression symptoms to reach reliable improvement and recovery, ** Recovery = reduction of depression and anxiety symptoms to a level below caseness (PHQ-9 < 10 and GAD-7 < 8), reliable improvement = reduction in depression symptom score and/or anxiety symptom score greater than reliable change index (PHQ-9 ≥ 6 and/or GAD-7 ≥ 4) or reduction in depression symptom score or anxiety symptom score without reliable deterioration on the other, reliable recovery = reliable improvement and recovery.

Supplementary Figure E4.1.1

Bootstrapped mean differences in costs and PHQ-9 scores at 34-week follow-up (health and social care perspective, complete case)

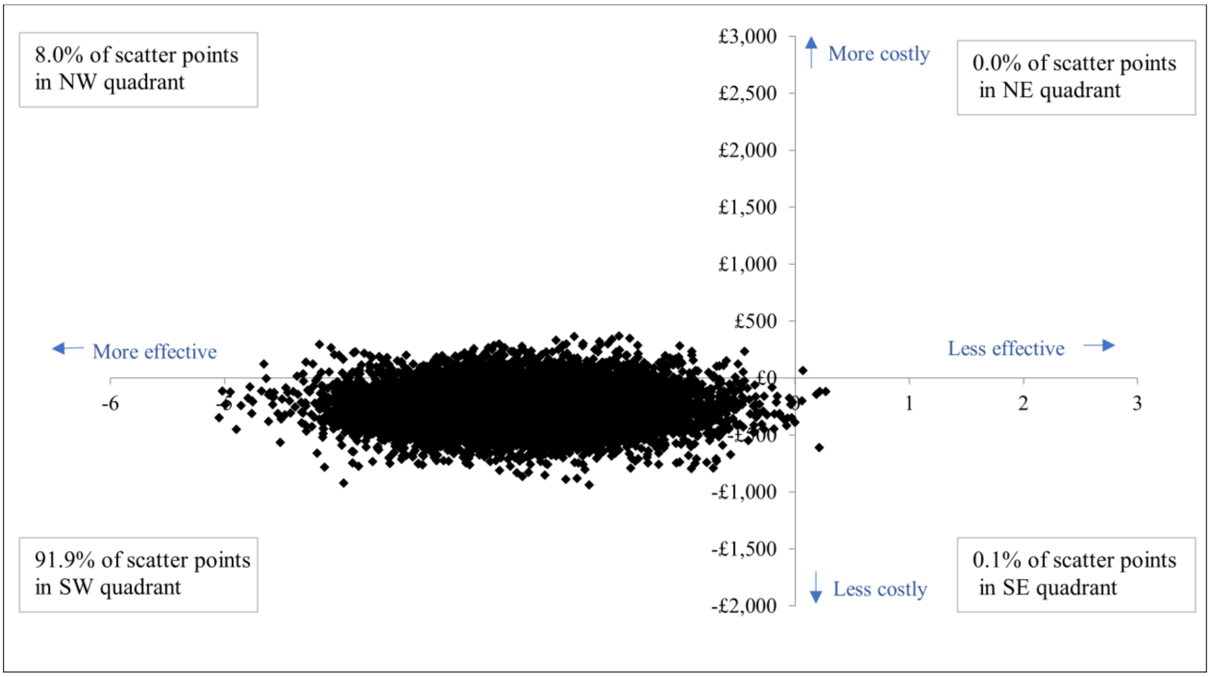

Based on 10,000 bootstrapped replications of adjusted model. NE, north-east (more costly, less effective); NW, north-west (more costly, more effective); SE, south-east (less costly, less effective); SW, south-west (less costly, more effective)

Supplementary Figure E4.1.2

Cost-effectiveness acceptability curve showing the probability that MBCT is cost-effective compared with TAU at different values of WTP thresholds per PHQ-9 score at 34-week follow-up (health and social care perspective, complete case)

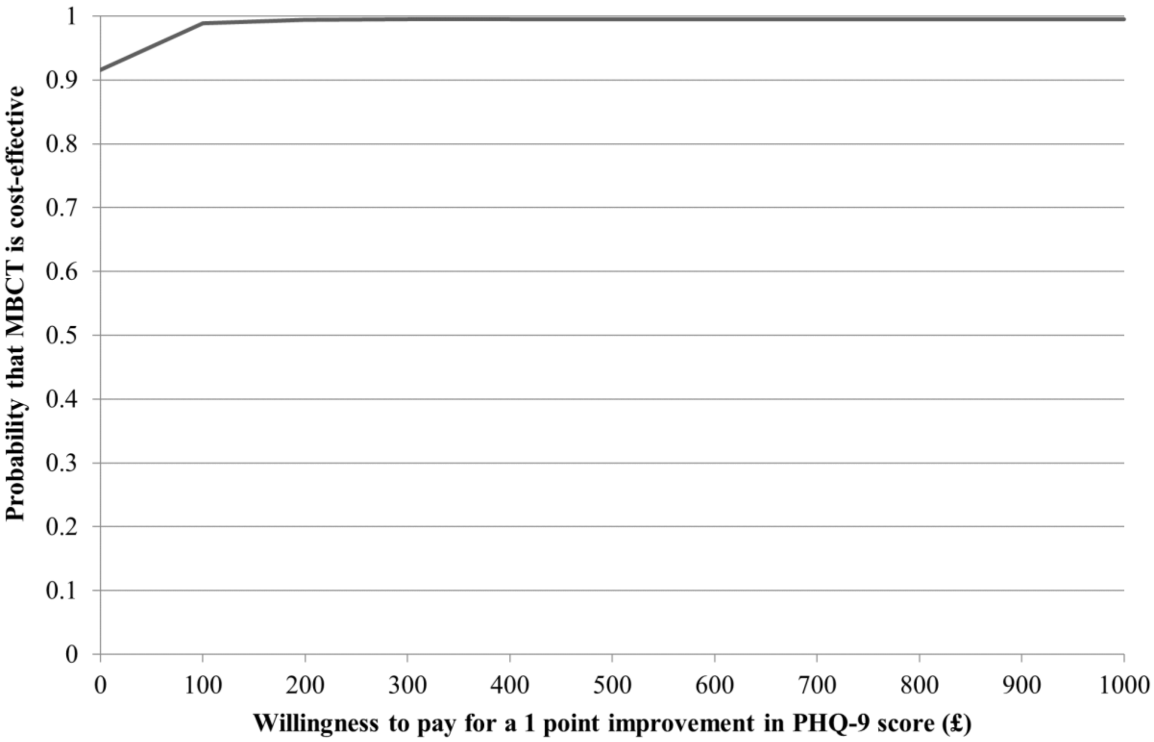

Supplementary Figure E4.1.3

Bootstrapped mean differences in costs and reliable recovery at 34-week follow-up (health and social care perspective, complete case)

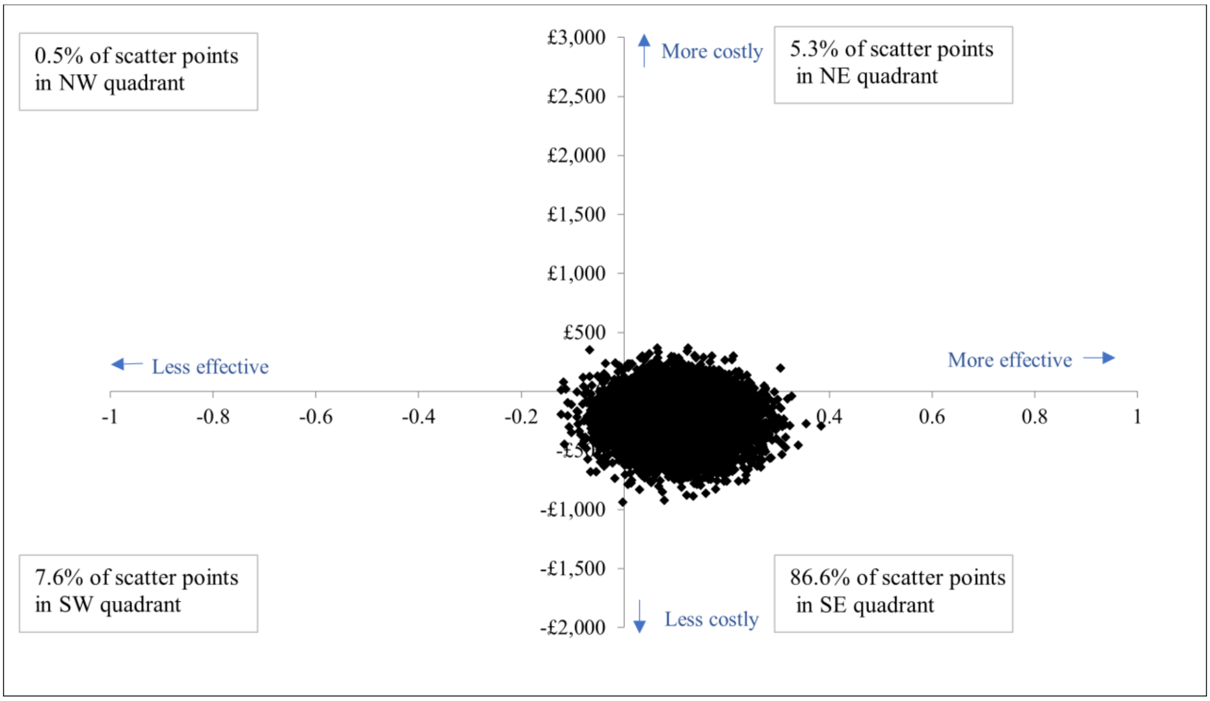

Figure E4.1.4

Cost-effectiveness acceptability curve showing the probability that MBCT is cost-effective compared with usual care at different values of WTP threshold to achieve reliable recovery 34-week follow-up: complete case health and social care perspective.

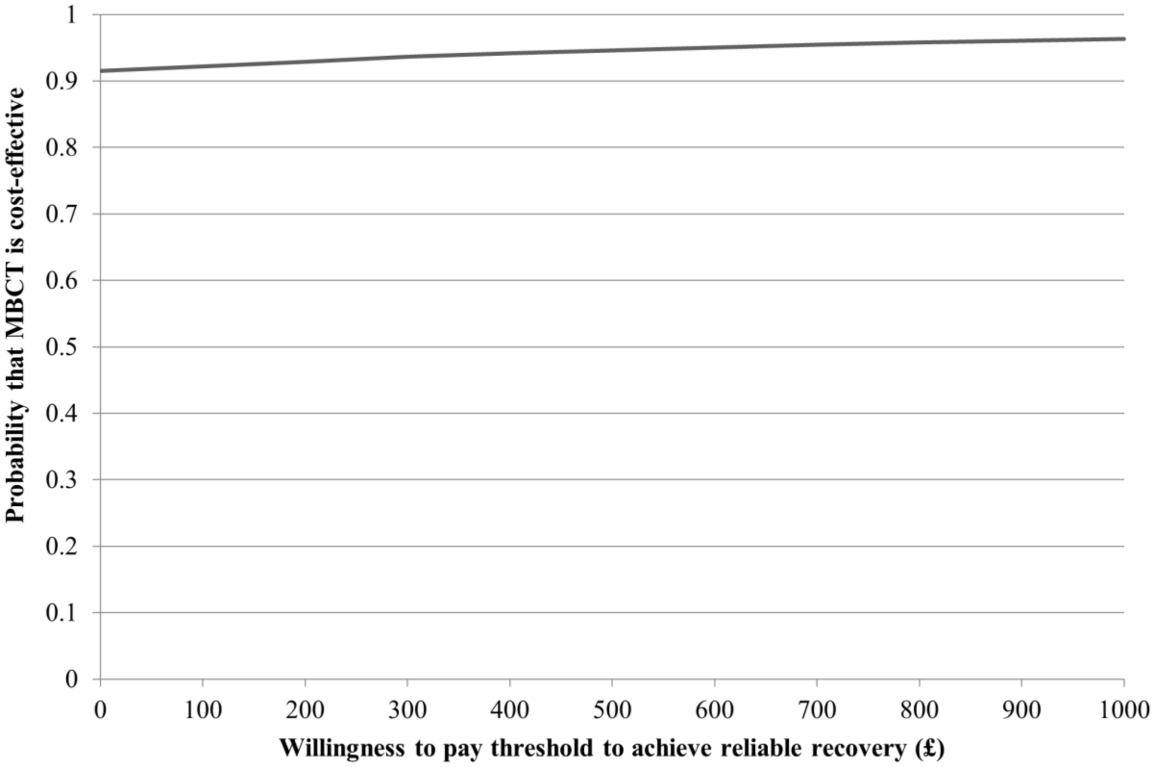

E4.2 Health and Social Care Perspective (Multiple Imputation)

Supplementary Table E4.2.1

Mean costs (£) per participant over 34 weeks follow-up (health and social care perspective, multiple imputation)

	Trial group, mean (SD)		MBCT minus TAU[a]			
**Cost category**	**MBCT**	**TAU**	**Unadjusted difference[b]**	**Adjusted difference[b,c]**	**95% CI**	**p-value**
**Baseline**						
Total	463·35 (789·28)	624·26 (1053·33)	-160·90	-158·19	-397·80 to 81·43	0·20
**34-week follow-up**						
MBCT intervention	62·86 (25·55)	0 (0)	62·86	62·87	58·21 to 67·52	<0·0001
Hospital services	385·69 (824·28)	638·94 (1,031·32)	-253·26	-228·40	-492·77 to 35·98	0·090
Community-based services	323·89 (454·83)	444·42 (494·50)	-120·53	-111·87	-240·17 to 16·43	0·087
Medication	98·64 (72·25)	97·41 (72·71)	1·23	6·55	-10·27 to 23·36	0·44
**Total**	871·09 (1,099·25)	1,180·78 (1,298·25)	-309·70	-255·07	-578·46 to 68·31	0·12

[a] Comparison based on multiple imputation data, [b] Unadjusted and adjusted differences based on 1,000 bootstrapped replications, [c] Adjusted for depression severity (PHQ-9 score <19 vs ≥19), antidepressant use at baseline, recruitment site and baseline costs

Supplementary Table E4.2.2

Mean utility scores and QALYs per participant (health and social care perspective, multiple imputation)

	Trial group, mean (SD)		MBCT minus TAU[a]			
**Quality of life**	**MBCT**	**TAU**	**Unadjusted difference[b]**	**Adjusted difference[b,c]**	**95% CI**	**p-value**
Baseline (utility)	0.517 (0.276)	0.504 (0.279)	0.013	0.008	-0.061 to 0.077	0.81
10 weeks (QALYs)	0.103 (0.049)	0.095 (0.051)	0.008	0.006	0.001 to 0.010	0.021
34 weeks (QALYs)	0.361 (0.168)	0.322 (0.176)	0.040	0.033	0.005 to 0.061	0.020

[a] Comparison based on multiple imputation data, [b] Unadjusted and adjusted differences based on 1,000 bootstrapped replications, [c] Adjusted for depression severity (PHQ-9 score <19 vs ≥19), antidepressant use at baseline, recruitment site and baseline utility

Supplementary Table E4.2.3

Mean PHQ-9 scores per participant applied to the cost-effectiveness analysis (health and social care Perspective, multiple imputation)

	Trial group, mean (SD)		MBCT minus TAU			
**PHQ-9 time point**	**MBCT**	**TAU**	**Unadjusted difference**	**Adjusted difference**	**95% CI**	**p-value**
Baseline	17.95 (3.92)	17.77 (3.83)	0.18	0.26	-0.292 to 0.814	0.35
34 weeks	12.79 (5.54)	14.90 (5.53)	-2.10	-2.24	-3.646 to -0.841	0.002

E5. Health and Social Care and Productivity Costs Perspective

E5.1 Health and Social Care and Productivity Costs Perspective (Complete Case)

Supplementary Table E5.1.1

Mean costs (£) per participant over 34 weeks follow-up (health and social care and productivity costs perspective, complete case)

	Trial group, Mean (SD)		MBCT minus TAU[a]			
**Cost category**	**MBCT**	**Usual care**	**Unadjusted difference[b]**	**Adjusted difference[b,c]**	**95% CI**	**p-value**
**Baseline**						
Total	663.08 (1,516.32)	904.26 (1,059.09)	-241.18	-250.99	-650.07 to 148.09	0.22
**10-week follow-up**						
Hospital services	114.31 (290.85)	180.40 (395.31)	-66.08	-47.06	-146.86 to 52.73	0.36
Community-based services	113.76 (197.90)	128.19 (257.20)	-14.43	-10.72	-76.30 to 54.85	0.75
Medication	25.54 (22.81)	31.09 (22.53)	-5.55	-1.02	-5.34 to 3.30	0.64
Missed employment	180.83 (857.36)	174.04 (460.80)	6.79	46.01	-165.99 to 258.01	0.67
Total	434.45 (1,064.31)	513.72 (734.93)	-79.27	1.95	-274.44 to 278.34	0.99
**34-week follow-up**						
MBCT intervention	67.72 (20.02)	0	67.72	67.77	63.69 to 71.85	<0.0001
Hospital services	383.40 (830.49)	647.85 (1,042.67)	-264.46	-230.37	-497.04 to 36.30	0.090
Community-based services	340.93 (474.59)	457.09 (506.03)	-116.15	-112.29	-248.65 to 24.08	0.11
Medication	96.62 (73.24)	99.81 (72.25)	-3.18	11.06	-2.67 to 24.80	0.11
Missed employment	518.00 (2,334.52)	511.05 (1,204.33)	6.95	138.21	-465.44 to 741.86	0.65
**Total**	1,406.66 (2,843.78)	1,715.80 (1,910.72)	-309.29	-99.75	-840.05 to 640.54	0.79

[a] Comparison based on complete case data, [b] Unadjusted and adjusted differences based on 5,000 bootstrapped replications, [c] Adjusted for depression severity (PHQ-9 score <19 vs ≥19), antidepressant use at baseline, recruitment site and baseline costs

Supplementary Table E5.1.2

Mean utility scores and QALYs per participant (health and social care and productivity costs perspective, complete case)

	Trial group, mean (SD)		MBCT minus TAU[a]			
**Quality of life**	**MBCT**	**TAU**	**Unadjusted difference[b]**	**Adjusted difference[b,c]**	**95% CI**	**p-value**
Baseline (utility)	0.524 (0.265)	0.512 (0.271)	0.012	0.009	-0.063 to 0.082	0.80
10 weeks (utility)	0.559 (0.272)	0.491 (0.288)	0.068	0.062	0.009 to 0.115	0.022
34 weeks (utility)	0.568 (0.289)	0.504 (0.310)	0.064	0.564	-0.004 to 0.117	0.069
10 weeks (QALYs)	0.104 (0.047)	0.096 (0.051)	0.008	0.006	0.001 to 0.011	0.022
34 weeks (QALYs)	0.364 (0.166)	0.326 (0.180)	0.038	0.033	0.005 to 0.061	0.020

[a] Comparison based on complete case data, [b] Unadjusted and adjusted differences based on 5,000 bootstrapped replications, [c] Adjusted for depression severity (PHQ-9 score <19 vs ≥19), antidepressant use at baseline, recruitment site and baseline utility

Supplementary Table E5.1.3

Mean PHQ9 scores per participant (health and social care and productivity costs perspective, complete case)

	Trial group, mean (SD)		MBCT minus TAU			
**PHQ-9 time point**	**MBCT**	**TAU**	**Unadjusted difference[b]**	**Adjusted difference[b,c]**	**95% CI**	**p-value**
Baseline	18.18 (3.92)	17.45 (3.72)	0.728	0.504	-0.105 to 1.114	0.10
10 weeks	13.98 (5.29)	15.75 (4.79)	-1.769	-2.283	-3.490 to -1.076	<0.0001
34 weeks	12.67 (5.52)	14.56 (5.48)	-1.888	-2.374	-3.843 to -0.905	0.002

[a] Comparison based on complete case data, [b] Unadjusted and adjusted differences based on 5,000 bootstrapped replications, [c] Adjusted for depression severity (PHQ-9 score <19 vs ≥19), antidepressant use at baseline, recruitment site and baseline PHQ-9 score

E5.2 Health and Social Care and Productivity Costs Perspective (Multiple Imputation)

Supplementary Table E5.2.1

Mean costs (£) per participant baseline and over 34-week follow-up (health and social care and productivity costs perspective, multiple imputation)

	**Trial group, mean (SD)**		**MBCT minus TAU[a]**			
**Cost category**	**MBCT (N=118)**	**TAU(N=116)**	**Unadjusted difference**	**Adjusted difference[b]**	**95% CI**	**p-value**
**Baseline**	684.89 (1,155.37)	949.20 (1,653.36)	-264.32	-259.31	-625.35 to 106.73	0.16
**34-week follow-up**						
MBCT intervention	62.86 (25.55)	0	62.86	62.86	58.21 to 67.53	<0.0001
Hospital services	394.53 (846.23)	630.10 (1,013.95)	-235.57	-208.24	-471.68 to 55.18	0.12
Community-based services	328.53 (458.84)	453.67 (506.48)	-125.14	-116.14	-248.84 to 16.57	0.086
Medication	100.23 (72.33)	97.36 (72.70)	2.86	8.30	-6.58 to 23.17	0.23
Missed employment	527.38 (2,330.71)	495.70 (1,163.10)	32.19	80.28	-463.82 to 540.07	0.91
Total	1,413.78 (2829.56)	1,676.56 (1,853.81)	-262.78	-141.78	-804.88 to 521.32	0.67

[a] Comparison based on multiple imputation data, [b] Adjusted for depression severity (PHQ-9 score <19 vs ≥19), antidepressant use at baseline, recruitment site and baseline costs

Supplementary Table E5.2.2

Mean baseline utility score and QALYs per participant (health and social care and productivity costs perspective, multiple imputation)

	**Trial group, mean (SD)**		**MBCT minus TAU[a]**			
**Quality of life**	**MBCT**	**TAU**	**Unadjusted difference**	**Adjusted difference[b]**	**95% CI**	**p-value**
Baseline (utility)	0.516 (0.276)	0.504 (0.279)	0.013	0.008	-0.061 to 0.078	0.81
10 weeks (QALYs)	0.104 (0.049)	0.095 (0.050)	0.007	0.006	0.001 to 0.011	0.017
34 weeks (QALYs)	0.362 (0.167)	0.323 (0.175)	0.040	0.033	0.006 to 0.060	0.020

[a] Comparison based on multiple imputation data, [b] Adjusted for depression severity (PHQ-9 score <19 vs ≥19), antidepressant use at baseline, recruitment site and baseline utility

Supplementary Table E5.2.3

Mean PHQ-9 scores per participant applied to the cost-effectiveness analysis (health and social care and productivity costs perspective, multiple imputation)

	Trial group, mean (SD)		MBCT minus usual care[a]			
**PHQ9 time point**	**MBCT**	**Usual care**	**Unadjusted difference**	**Adjusted difference[b]**	**95% CI**	**p-value**
Baseline	17.95 (3.92)	17.76 (3.82)	0.18	0.26	-0.292 to 0.815	0.35
34 weeks	12.78 (5.49)	14.87 (5.51)	-2.10	-2.24	-3.65 to -0.84	0.002

[a] Comparison based on multiple imputation data, [b] Adjusted for depression severity (PHQ-9 score <19 vs ≥19), antidepressant use at baseline, recruitment site and baseline PHQ-9 score

F. Modifications to Original MBCT Treatment Manual

Delivery of the MBCT intervention followed the second edition of the MBCT manual (Segal, Williams & Teasdale, 2013) with minor formal adaptations to address the fact that participants were currently depressed rather than in remission. Within the formal structure of MBCT, therapists responded flexibly to the current state of participants in line with usual practice for enquiries and psycho-educational teaching. They were instructed to carefully monitor signs for emotional contagion within the group and invited to use these as opportunities for recognising unhelpful patterns of thinking. Teachers were instructed to embody a mindful approach as usual, but to be prepared to be more proactive in structuring the sessions, for example by deliberately using changes between larger group and small group enquiries (i.e. videoconferencing breakout rooms) and by leading the enquiries at a pace that counters fatigue where necessary. Participants practiced with the guided meditation recordings available with the treatment manual.

Session 1: **Awareness and Automatic Pilot**

The introduction highlighted that the main aim of the course is learning to approach experience, including difficult thoughts and feelings, from a different angle. It was emphasised that such changes in perspective are likely to be particularly helpful when patients are experiencing negative mood, although it was acknowledged that it might feel like skills are more difficult to apply under this condition. The introduction highlighted the need for regular practice while emphasising the importance of compassion towards oneself in situations where good intentions may be difficult to follow. The structure, content, practices (body scan) and exercises (raisin exercise) of the session remained unchanged.

Rationale

- Mindfulness starts when we recognise the tendency to be on automatic pilot
- Commitment to learning how to step out of it and become aware of each moment
- Practice in purposefully moving attention round the body

Practice

- Eating meditation (Raisin)
- Body scan
- Routine activity (homework)
- ‘Noticing’ (homework – continues throughout)

Session 2: **Living in Our Heads**

Structure, content, practices (body scan, sitting meditation) and exercises (thoughts and feelings exercise) of the session remained unchanged. The homework instruction for the pleasant experiences diary explicitly asked participants to look out for small changes in mood and encouraged patients to be become aware of small fluctuations in mood to prevent demoralisation in case of dominant negative mood.

Rationale

- Further focus on the body begins to show more clearly the chatter of the mind
- This chatter tends to control our reactions to everyday events: Situation + Interpretation ⇨ Emotion, body sensations, behaviour (thoughts are not facts)

Exercises

- o Thoughts and feelings
- o Focus on pleasant events (homework)

Practice

- o Body scan
- o Breath
- o Routine activity (homework)

Session 3: **Gathering the Scattered Mind**

It was recommended that the teachers followed the alternative structure offered in the manual for this session, which emphasises movement practices and starts with a long mindful movement sequence. However, teachers were free to decide against this recommendation based on their own reading of the needs of the participants in their group.

Rationale

- o Becoming familiar with the behaviour of the mind (often be busy and scattered)
- o Awareness of the breath offers a possibility of being more focussed and gathered (an anchor to the present moment)
- o Categorising experiences vs. describing bare sensations
- o Mindful movement: Yoga, stretching, walking

Exercises

- Focus on unpleasant events (homework)

Practice

- Seeing/hearing
- Breath and body
- 3-minute breathing space
- Stretch and breath/mindful movement(homework)

Session 4: **Recognizing Aversion**

Participants were guided through the exercise aimed at recognising the negative automatic thoughts as the 'voice of depression' as outlined in the manual. Teachers were made aware that in acute depression, participants might find it more difficult to identify biased thinking and asked to aid identification through dialogue. They were instructed to clearly anchor the exercise in a past situation where participants felt depressed and to highlight discrepancies between this situation and a past situation where patients felt well. Teachers used changes in mood following the reading of the list of automatic thoughts as an opportunity to recognise the dynamics of negative thinking and followed the manual by guiding patients to respond to such changes with a breathing space. This part of the exercise was deliberately used as an opportunity for exposure and teachers were instructed to pace the exercise in a way that minimises distractions. The structure, content, practices (hearing meditation, sitting meditation, mindful walking) and exercises recognising the territory of depression) of the session remained unchanged.

Rationale

- The mind is most scattered when trying to cling to some things and avoid others
- Mindfulness offers a way to stay present by providing another place from which to view things
- To help take a wider perspective/relate differently to experience (including thoughts/feelings)
- Getting to know the territory of depression

Exercises

- Defining the territory of depression (ATQ, DSM criteria)

Practice

- Seeing/hearing meditation
- Breath, body, sounds, thoughts, choiceless awareness
- Mindful walking

Session 5: **Allowing/Letting Be**

Structure, content, practices (sitting with the difficult meditation) and exercises of the session remained unchanged. Teachers were asked to be particularly vigilant for signs that participants might be experiencing traumatic memories and provided additional information on how to work with intrusive imagery.

Rationale

- Relating differently involves bringing to experience a sense of allowing it to be as it is, without judging it or trying to make it different
- Such an attitude of acceptance is a major part of taking care of oneself and seeing clearly what, if anything, needs to change

Practice

- Breath, body, difficulty
- Expanded breathing space

Session 6: **Thoughts are Not Facts**

Structure, content, practices (sitting meditation) and exercises of the session remained unchanged. The relapse signature exercise was introduced with an understanding that levels of symptoms fluctuate over time so that it was accessible even for patients who were suffering from long and persistent episodes of depression.

Rationale

- Negative moods and thoughts that accompany them colour our ability to relate to experience
- It is liberating to realise that our thoughts are merely thoughts – we can choose whether to engage with them
- Thoughts are just thoughts – even the ones that say they are not
- Recognising that the same patterns of thought recur again and again can help us to stand back from our thoughts, without necessarily having to question them and seek alternatives

Exercises

- Alternative perspectives (the office photocopier)
- Options for working with thoughts
- Relapse signatures

Practice

- Breath, body, sounds, thoughts and emotions, difficulty, choiceless awareness
- Breathing space
- Selection of practices (homework)

Session 7: Structure, content, practices (sitting meditation) and exercises of the session remained unchanged. Teachers emphasised the potential of activity in current episodes of depression based on models of behavioural activation.

Rationale

- Specific things can be done when depression threatens
- Take a breathing space first, and then decide what action (if any) to take
- Each person has his or her own unique relapse signature
- Participants in the classes can help each other to plan how best to respond to the signs

Exercises

- Activity and mood (nourishing vs. depleting activities)
- Plan how best to schedule activities for when mood threatens to overwhelm
- Identifying actions to deal with threat of relapse

Practice

- Breath, body, noticing how we relate to experiences
- Breathing space
- Selecting forms of practice you will be able to continue with (homework)

Session 8: **Maintaining and Extending Learning**

Structure, content, practices (sitting meditation) and exercises of the session remained unchanged.

Rationale

- Maintaining balance in life is helped by regular mindfulness practice
- Good intentions can be strengthened by linking the practice with + reasons for taking care of oneself

Exercises

- What are the things in your life that you most value, that the practice could help you with?
- Preparing for the future (action plans)

Practice

- Body scan
- Final sitting, well-wishing

Programme Delivery

The group sessions were delivered via videoconference using a secure online platform (Zoom). Delivery followed suggestions on how key features of mindfulness-based teacher competence should be represented in online delivery outlined in the "Addendum to the Mindfulness-Based Interventions: Teaching Assessment Criteria (MBI:TAC) for assessing online delivery of mindfulness-based programs" (Sansom et al., 2020). Videoconference delivery comes with advantages in terms of reach and scalability. However, there are also potential restrictions including the fact that this delivery format requires access to and expertise in using relevant IT equipment, which may pose problems for elderly or economically disadvantaged participants.

Sansom, S., Crane, R., Karunavira, Koerbel, L., & Yiangou, A. (2020). Addendum to the Mindfulness-Based Interventions: Teaching Assessment Criteria (MBI:TAC) for assessing online delivery of mindfulness-based programs. https://mbitac.bangor.ac.uk/documents/addendum-online-delivery.pdf

Segal ZV, Williams JMG, Teasdale JD. Mindfulness-Based Cognitive Therapy for Depression: Second Edition. New York: Guilford Press; 2013.

G. Serious Adverse Events

Seven serious adverse events were recorded over the duration of the trial (two in MBCT+TAU, three in TAU and three in participants who had not been randomised), all of which were deemed to be unrelated to the trial and intervention by the independent clinical monitor. Detailed descriptions of the SAEs are provided in supplementary table G1.

Supplementary Table G1

Serious adverse events over the duration of the trial

Site	Visit	Classification of SAE	Nature of the event	Date reviewed by independent clinical monitor	Gender
Sussex	Screening	Life threatening	During a clinical assessment (MINI) a participant was screened for risk of suicidality. The participant disclosed the day prior to the screening they had contemplated taking their own life.	04.10.2021	Male
London	34-week FU	Required In-patient hospitalisation or prolongation of existing hospitalisation	Whilst completing the AD-SUS questionnaire of the 34-week FU, the participant disclosed that they had spent 2 nights overnight on a medical/surgical/general ward for “weight loss surgery”.	23.08.2022	Female
London	34-week FU	Required In-patient hospitalisation or prolongation of existing hospitalisation	Whilst completing the AD-SUS questionnaire of the 34-week FU, participant disclosed that they had spent 1 night on a medical/surgical/general ward as a precaution following a planned procedure (“gallbladder removal”)	23.08.2022	Male
Devon	Screening	Other important event- presented to A&E during the day	Participant disclosed they were in A&E when called for MINI assessment due to blistering after accidentally dropping a pan of boiling water.	10.10.2022	Female
Devon	34-week FU	Required In-patient hospitalisation or prolongation of existing hospitalisation	Participant disclosed they were in hospital after being diagnosed with three different infections.	30.01.2023	Female
Sussex	34-week FU	Required In-patient hospitalisation or prolongation of existing hospitalisation	Participant experienced heart problems and following investigative appointments underwent emergency surgery to have stints fitted.	25.09.2023	Male
Sussex	34-week FU	Required In-patient hospitalisation or prolongation of existing hospitalisation	Participant sought treatment for a Urinary Tract Infection and was admitted to hospital with suspected sepsis.	06.10.2023	Prefer not to say

H. Internal Consistency of Outcome Measures

Supplementary Table H1

Internal consistency of outcome measures (as assessed at baseline)

Measure	Cronbach's α	McDonald's Ω
**PHQ-9**	0·662	0·642
**GAD-7**	0·834	0·833
**Phobia Scale**	0·858	0·868
**WSAS**	0·749	0·775
**WEMWBS**	0·811	0·807
**FFMQ**	0·733	0·676
**EQ Decentering**	0·793	0·788

I. Patient Identification Centres (PIC)

Supplementary Table I1

Patient Identification Centres

Affiliated site	PIC site
1	Devon Partnership NHS Foundation Trust
1	Dorset Healthcare University NHS Foundation Trust
1	Gloucestershire Health and Care NHS Foundation Trust
1	Cornwall Partnership NHS Foundation Trust
1	Somerset NHS Foundation Trust
2	Sussex Partnership NHS Foundation Trust (East Sussex, West Sussex)
2	Sussex Community NHS Foundation Trust
2	Surrey and Borders Partnership NHS Foundation Trust
2	North East London NHS Foundation Trust (NELFT)
2	Hertfordshire Partnership University NHS Foundation Trust
2	Coventry & Warwickshire Partnership Trust
3	South London and Maudsley NHS Foundation Trust (Southwark Talking Therapies, Lambeth Talking Therapies, Lewisham Talking Therapies, Croydon Talking Therapies),
3	Berkshire Healthcare NHS Foundation Trust – Talking Therapies IAPT Service
3	Nottinghamshire Healthcare NHS Foundation Trust
3	Lincolnshire Partnership NHS Foundation Trust

1 = Devon, 2 = Sussex, 3 = London.

J. PPI Involvement

From its inception, the involvement of people affected by depression has been integral to this research. The research question was derived from a scoping exercise conducted with former patients of the Mood Disorders Centre Clinic in Exeter, in which participants identified areas in which future research could help to improve services for depressed patients. The project and study design was further discussed in meetings with the Lived Experience Group at the Mood Disorder Centre and refined to address gaps in the current provision of standard services. MR joined as part of the research team and PPI lead following these discussions, bringing personal experience of depression. She was part of the team that conducted a feasibility study, working closely with other researchers to understand themes emerging from qualitative analysis and consider possible modifications to the therapy. She provided a PPI perspective to the writing of the grant proposal for the current study, served as a co-applicant and led the patient advisory group of three experts by lived experience, which was regularly consulted during the conduct of the study. The group provided invaluable advise on procedures for recruitment, assessment including the mitigation of burden created by questionniares and safe delivery of the interventions including work on preventing 'resentful demoralisation' in the TAU only arm of the study. It held a sense-making workshop shortly after results of the study were available, the outcomes of which have critically informed the interpretation and dissemination of study findings. MR is a co-author of the current report and has been an integral part of all dissemination efforts so far, including as a discussant of findings at conferences and workshops.

Throughout this document the following acronyms are used:

MBCT: Mindfulness-Based Cognitive Therapy

TAU: Treatment as usual
